# Longitudinal changes in reproductive hormones through the menopause transition in the Avon Longitudinal Study of Parents and Children (ALSPAC)

**DOI:** 10.1038/s41598-020-77871-9

**Published:** 2020-12-04

**Authors:** Ana Goncalves Soares, Fanny Kilpi, Abigail Fraser, Scott M. Nelson, Naveed Sattar, Paul I. Welsh, Kate Tilling, Deborah A. Lawlor

**Affiliations:** 1 MRC Integrative Epidemiology Unit at the University of Bristol, Bristol, UK; 2 Population Health Science, Bristol Medical School, University of Bristol, Oakfield House, Oakfield Grove, Bristol, BS8 2BN UK; 3 Bristol NIHR Biomedical Research Centre, Bristol, UK; 4 School of Medicine, Dentistry and Nursing, University of Glasgow, Glasgow, UK; 5 Institute of Cardiovascular and Medical Sciences, University of Glasgow, Glasgow, UK

**Keywords:** Epidemiology, Risk factors

## Abstract

We characterised changes in reproductive hormones—LH, FSH, SHBG and AMH—by chronological age and time around the menopause (reproductive age) in mid-life women and explored their associations with lifestyle and reproductive factors. We used data from 1608 women from a UK cohort who had repeat hormone measures and experienced a natural menopause. Multilevel models were used to assess: (i) changes in hormones (outcomes) by reproductive age and chronological age (these age variables being the key exposures) and (ii) associations of body mass index (BMI), smoking, alcohol intake, parity and age at menarche with changes in hormones by reproductive age. Both LH and FSH increased until ~ 5 and 7 years postmenopause, respectively, after which they declined, but not to premenopausal levels. SHBG decreased slightly until ~ 4 years postmenopause and increased thereafter. AMH decreased markedly before menopause and remained low subsequently. For all hormones, the best fitting models included both reproductive and chronological age. BMI, smoking and parity were associated with hormone changes; e.g., higher BMI was associated with slower increase in LH and FSH and decrease in AMH. Reproductive and chronological age contribute to changes in LH, FSH, SHBG and AMH across mid-life in women, and BMI, smoking and parity are associated with these hormone changes.

## Introduction

Cross-sectional and longitudinal studies suggest that age at menopause is associated with numerous health outcomes, including inverse associations with adverse cardiometabolic, skeletal and mental health outcomes^1–4^. Among other factors, it is assumed that these associations might be driven, at least to some extent, by the hormonal changes that occur around the time of the menopausal transition^5–7^, but these changes are not well characterised for some reproductive hormones.

There is evidence that levels of follicle-stimulating hormone (FSH) and luteinizing hormone (LH) increase with reproductive age, particularly from 2-years before to 2-years after the final menstrual period (FMP), and thereafter remain at the same high levels^8–10^. Most longitudinal studies suggest that sex-hormone binding globulin (SHBG) levels decrease during the menopausal transition^11–13^, though one study showed an increase^14^. Anti-Müllerian hormone (AMH) declines before the FMP and has been extensively explored as a predictor of time to menopause^15–17^. Whilst these studies report changes in AMH levels with chronological age, few longitudinal studies have described changes of AMH levels with reproductive age. Where this has been described, results suggest that the pattern of decline is not uniform between women, and varies depending on pre-menopausal AMH levels, with women who had higher AMH level 20 years before the FMP having a slower decline between 20 and 15 years before the FMP and reversing to a faster decline in the last 5 years before the menopause^15^. Most studies with repeatedly assessed hormone data tend to have small sample sizes and do not compare whether change over time is best predicted by reproductive or chronological ageing.

Several studies have examined associations of lifestyle and reproductive factors with single measure concentrations of LH, FSH, SHBG and AMH^18–24^. Fewer longitudinal studies have assessed how lifestyle (e.g. body mass index (BMI), smoking, alcohol intake, physical activity) and earlier reproductive factors (e.g. parity and age at menarche) are associated with hormonal changes. In Supplementary Table 1 we summarise the seven studies we identified in a literature search that explore the associations of lifestyle and reproductive factors with change in reproductive hormones in women in mid-life^8,9,16,25–28^.

The aims of this study were to (1) characterise the patterns of change in FSH, LH, SHBG, and AMH through the menopausal transition; (2) determine whether these change patterns are associated with reproductive age (i.e. time around the final menstrual period) or chronological age, or both; and (3) explore associations of lifestyle and reproductive factors with these hormone patterns.

## Methods

### Participants

Data from the Avon Longitudinal Study of Parents and Children (ALSPAC) were used. All pregnant women resident in Avon, a former county comprising the area surrounding the city of Bristol, United Kingdom (UK), who had an estimated delivery date between 1 April 1991 and 31 December 1992 were eligible for the study, and 14,451 pregnancies were enrolled^29^. Pregnant women originally resident in Avon but migrating out of the catchment area prior to delivery were excluded, unless they had completed the questionnaire scheduled for the third trimester of pregnancy. Full details of recruitment, follow-up and data collection for these women, as well as their children and partners, have been reported elsewhere^29,30^, and the study website contains details of all the data that are available through a fully searchable data dictionary and variable search tool (http://www.bristol.ac.uk/alspac/researchers/our-data/). Ethical approval for the ALSPAC Study was obtained from the ALSPAC Law and Ethics Committee and UK National Health Service Research Ethics Committees. All procedures performed were in accordance with the ethical standards of the ALSPAC Law and Ethics. Participants provided written informed consent regarding their participation.

**Figure 1:**
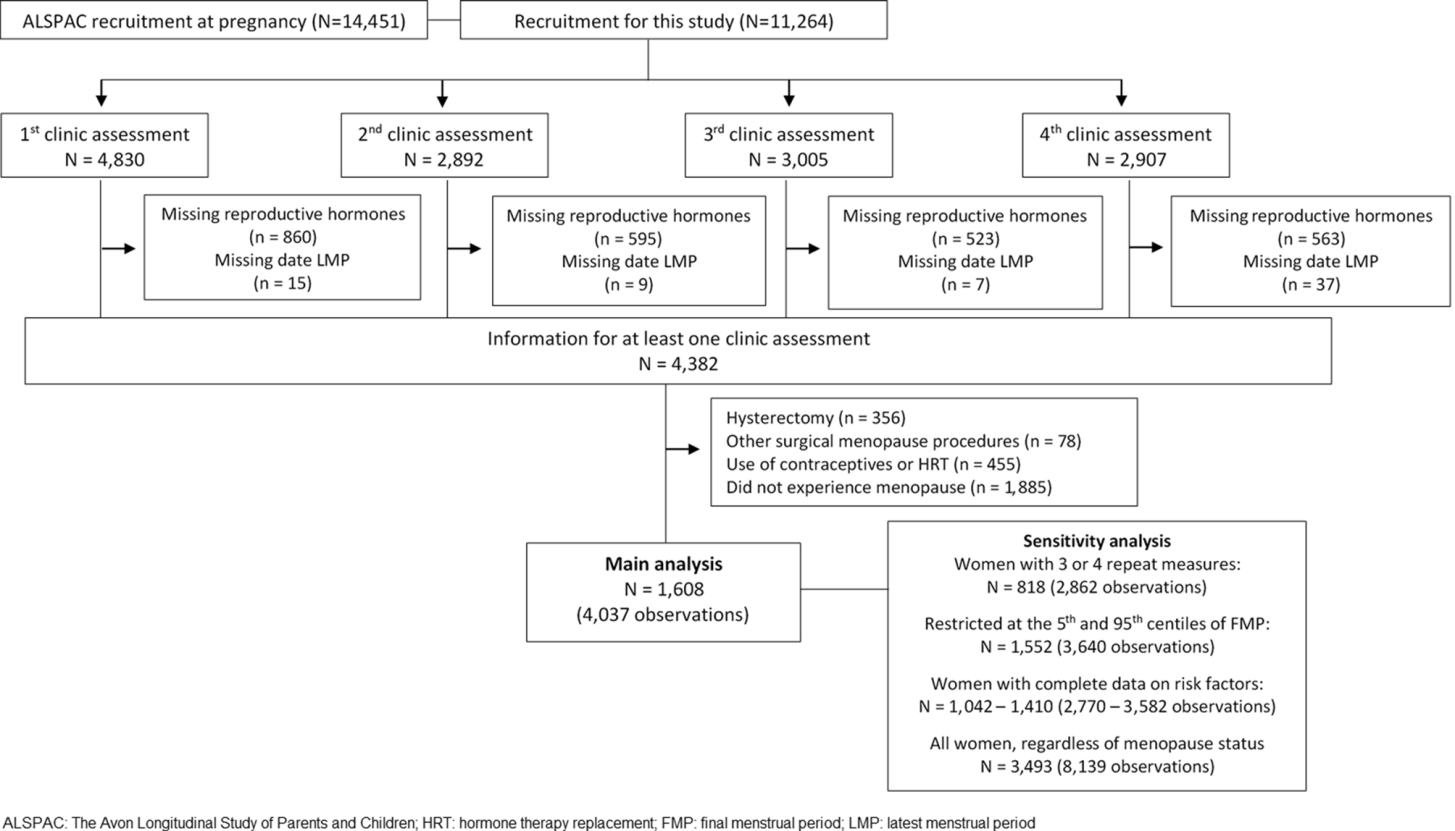
Participant flow into analysis groups.

Approximately 18 years after enrolment in ALSPAC (where enrolment was during the “index” pregnancy), all mothers still engaged with ALSPAC (N = 11,264) were invited to join this study, which included detailed clinic assessments repeated up to four times^29,31^. Women who were pre- or perimenopausal at the first clinic assessment and aged 40 years or over, and therefore likely to make a transition through one or more stages of the menopausal transition in the following 5 years, were invited to attend the further three clinic assessments^29^. The assessments were completed as follows: first, between 2009 and 2011 when women were [median (IQR)] aged 48 (45; 51); second, about 2.5 years later, between 2011 and 2013 when aged 51 (48, 54); third, approximately 1.3 years later, at 2013–2014 when aged 52 (49, 55); and fourth, about 1 year later, at 2014–2015 when aged 53 (50, 56). Women were included in the specific analyses presented here if they attended at least one of these clinic assessments, had data on reproductive hormones and had experienced the menopause (at least 12 months with no menstrual periods) by the last clinic assessment. As we were interested in patterns of change in hormone levels (our outcomes) related to a natural menopausal transition, we excluded women who had experienced any of the following: hysterectomy, oophorectomy, endometrial ablation, or radio- or chemotherapy related to reproductive organs. Observations from women reporting using hormonal contraception or hormone replacement were censored at the measurement before starting hormones. Following these exclusions, our analysis included 1,608 women, with 4,037 measures (Fig. 1).

### Assessments of reproductive hormones

In all four assessments, blood samples were taken following a standardised protocol. After collection, the blood was immediately centrifuged and frozen at − 80 °C, and the samples were assayed within 3 years of storage with no previous freeze–thawing cycles. Levels of four reproductive hormones were assessed: FSH, LH, SHBG, and AMH. AMH was measured using the fully automated Elecsys AMH Plus immunoassay^32^. All other hormones were measured with a Roche Elecsys modular analytics Cobas e411 using an electrochemiluminescence immunoassay using the manufacturers calibrators and quality control material.

### Assessments of factors that might influence changes in reproductive hormones in mid-life

At each clinic assessment, women were asked a detailed set of questions about their menstrual cycle, the date of their last menstrual period and the frequency and regularity of their menses, as well as lifestyle information and collection of anthropometric measures^31^. For each woman, reproductive age was calculated by subtracting the date of her FMP from the date which she attended the clinic assessment. Reproductive age was represented in years before and after the date of her menopause, which was the date of the menstrual period reported before the absence of menses for 12 or more months; hence 0 is the date of her FMP, − 1 would be 1 year before that date and + 1, 1 year after that date. Therefore, reproductive age was only defined for women who went through the menopause during the study period. Stages of Reproductive Aging Workshop (STRAW) criteria were used to categorise women into one of three mutually exclusive reproductive stages at each clinic assessment: (i) pre-menopausal (late reproductive age); (ii) menopausal transition; and (iii) postmenopause (irrespective of the years since menopause)^31,33^.

As we wanted to explore prospective associations of risk factors with reproductive hormone patterns, we used measures for BMI, smoking status and alcohol intake that were obtained at, or before, the first clinic assessment. Information on recalled age at menarche, number of previous pregnancies, age at first pregnancy and maternal education were obtained around the time of recruitment to the study (mean age 28.3, SD 4.8), with information from subsequent questionnaires used to update parity (last data obtained at a similar time to the first mid-life clinic assessment when women were mean 48.4 (SD 4.4) years old).

### Statistical analyses

The analyses were performed in the software Stata 15.1 (Statacorp, College Station, TX, USA) and the software MLwiN 3.04, accessed from within Stata using *runmlwin*^34^. We log-transformed all the hormones to obtain approximately normally distributed residuals in regression models. We then back transformed results for ease of interpretation. We used multilevel models to examine associations of each hormone with chronological and reproductive ageing, allowing for repeated measures within women. The multilevel models include all women with at least one hormone measure, under the missing at random (MAR) assumption. We used fractional polynomials to assess non-linear associations^35^. More details on fractional polynomials are presented in supplementary material. The power functions in the models varied (details available in supplementary material). Hormone levels were modelled against reproductive age alone, chronological age alone and with both time scales in the model; in the latter, age was centred at 51 years (mean age in our study). Given time has to be greater than zero when using fractional polynomials, a constant of 5 was added to time since FMP (as the lowest time since FMP was − 4.7 years). Model fit was assessed using Akaike Information Criterion (AIC) and Bayesian Information Criterion (BIC). Average predicted means for each reproductive hormone by reproductive age and chronological age were calculated from the multilevel models.

To explore whether hormone levels differed by categories of risk factors, multilevel models (as above) for each hormone were fitted with an interaction term between the risk factor and reproductive age. The risk factor categories used in these analyses were: BMI (normal/underweight (≤ 24.9 kg/m^2^), overweight (25.0–29.9 kg/m^2^), obese (≥ 30.0 kg/m^2^)); smoking (never, past, current); alcohol intake (never or ≤ 4 times/month, 2–3 times/week, ≥ 4 times/week), age at menarche (early (≤ 11 years), average (12–14 years), late (≥ 15 years)), and parity (1, 2, 3, ≥ 4 pregnancies). These analyses were adjusted for age, educational achievement and other potential confounders. The latter varied by which factor was being associated with the outcomes (full details in supplementary material). Average predicted means for each reproductive hormone by reproductive age were calculated for each category of the risk factors.

We performed post hoc analysis exploring whether hormone changes by reproductive age differed by age at menopause. We divided age at menopause into quintiles and fitted multilevel models including an interaction term between the quintiles of age at menopause and reproductive age.

### Dealing with missing data

There were missing data on some of the potential risk factors and confounders, in particular for alcohol (31%) and smoking (8.5%) (Supplementary Table 2). Missing data were imputed using multivariable multiple imputation with chained equations, performed using the *mice* command in Stata^36^. We used 50 imputed data sets and included all variables included in any models (including the time-varying hormone measures) in the imputation models. Data on smoking status and alcohol intake from questionnaires completed up to 7 years prior to the first mid-life clinic assessment were also used in the prediction models for missing risk factors.

### Sensitivity analyses

To circumvent the possibility of influential outliers, we performed sensitivity analysis restricting the data at the 5th and 95th centiles of time since FMP (n = 1,552 women with 3,640 observations). To explore the sensitivity of our results to including all women with at least one hormone measure we also repeated analyses only in women with 3 or 4 repeat measures (n = 818 women with 2,862 observations). We compared our main analyses (using multivariable imputation for missing risk factor or confounder values) to analyses including only those with complete data on risk factors and confounders (n varying from 1,042 to 1,410 women with 2,770 to 3,582 observations). Our main analyses can only include women who are known to have gone through the menopause, as this is required to calculate reproductive age. This might introduce selection bias, and to explore this, we compared patterns of change in hormone levels by chronological age in all women, irrespective of whether or not they had gone through the menopause (n = 3,493 women with 8,139 observations), to the main results for patterns of change by chronological age (i.e. in only those who had gone through the menopause). An indicator variable of whether the woman experienced menopause (and therefore was included in the main analysis) or not (therefore excluded from the main analysis) was created. Since for those with unknown status of menopause the time since FMP could not be estimated, a constant of zero was used (i.e. we assumed those women to be premenopausal). The models for the sensitivity analyses were fitted with an interaction term between menopause (yes/no) and chronological age, adjusted for time since FMP.

We also performed post hoc sensitivity analysis using generalized estimating equations (GEE). GEE analyses assume a different pattern of missingness (missing completely at random—MCAR) than multilevel models and are more robust to the misspecification of the covariance structure. Additionally, using information from women with known and unknown date of menopause and with information on menopausal stages (n = 3,460 women with 8,021 observations), we performed GEE analysis of patterns of change in reproductive hormones by chronological age according to menopausal stages (premenopause, perimenopause and postmenopause); these models were fitted with an interaction term between the menopausal stages and chronological age.

## Results

Women included in the analysis were aged 37–61 years at the first assessment and 43–66 years at the last assessment. Mean (SD) age of menopause was 49.9 (3.7) years. The correlation between reproductive and chronological age was r = 0.63. Characteristics of the sample are presented in Tables 1 and 2. Overall, 50% of the women were normal weight, 55% were never smokers, 40% consumed no alcohol or consumed it up to 4 times/month, 11% had experienced only one pregnancy, 70% had an age at menarche between 12 and 14 years, and 26% had a university degree.

**Table 1:** Distribution of menopausal stage, age, body mass index (BMI) and reproductive hormones at each clinic (N = 1,608 women).

	1st assessment	2nd assessment	3rd assessment	4th assessment
	N = 1,321	N = 875	N = 945	N = 896
	N (%)	N (%)	N (%)	N (%)
**Menopausal stage**				
Premenopause	281 (21.3)	0	0	0
Perimenopause	493 (37.3)	322 (36.8)	167 (17.7)	4 (0.4)
Postmenopause	547 (41.4)	553 (63.2)	778 (82.3)	892 (99.6)
**Age (years): mean (SD)**				
All	51.0 (4.0)	53.6 (3.9)	54.8 (3.8)	56.0 (3.6)
Premenopause	48.3 (3.2)	–	–	–
Perimenopause	49.6 (3.3)	51.1 (3.3)	51.8 (3.5)	^a^
Postmenopause	53.6 (3.4)	55.0 (3.5)	55.5 (3.5)	56.1 (3.5)
**BMI (kg/m**^**2**^**): mean (SD)**				
All	25.8 (4.7)	25.7 (4.6)	25.6 (4.8)	25.8 (5.0)
Premenopause	25.8 (4.8)	–	–	–
Perimenopause	25.6 (4.6)	26.2 (4.9)	26.5 (5.3)	^a^
Postmenopause	25.9 (4.8)	25.5 (4.4)	25.4 (4.6)	25.6 (4.8)
**LH (mIU/ml): median (IQR)**				
All	30.7 (12.6; 44.8)	37.3 (28.2; 46.9)	40.1 (31.8; 50.2)	37.3 (30.2; 46.2)
Premenopause	10.2 (5.5; 24.9)	–	–	–
Perimenopause	24.9 (8.7; 42.2)	32.8 (17.8; 43.2)	39.5 (29.7; 50.3)	^a^
Postmenopause	40.3 (30.9; 49.3)	39.2 (31.5; 48.8)	40.3 (32.3; 49.6)	37.0 (30.5; 46.2)
**FSH (mIU/ml): median (IQR)**				
All	53.3 (14.2; 81.0)	69.8 (48.4; 91.2)	83.9 (65.5; 104.1)	82.2 (65.4; 101.4)
Premenopause	11.9 (5.7; 29.3)	–	–	–
Perimenopause	33.4 (9.9; 64.7)	50.0 (26.4; 73.2)	72.3 (48.5; 94.9)	^a^
Postmenopause	78.4 (59.8; 100.9)	77.6 (62.0; 97.0)	85.5 (68.4; 106.5)	82.7 (66.6; 101.7)
**SHBG (nmol/L): median (IQR)**				
All	63.1 (45.4; 86.8)	64.5 (47.9; 86.6)	71.4 (51.7; 94.4)	66.8 (47.4; 89.9)
Premenopause	63.5 (48.3; 88.8)	–	–	–
Perimenopause	67.8 (49.5; 88.8)	63.4 (45.0; 88.3)	68.8 (49.0; 91.2)	^a^
Postmenopause	59.9 (42.0; 81.5)	64.7 (48.8; 85.0)	71.7 (51.7; 95.0)	66.9 (47.5; 90.0)
**AMH (ng/ml): median (IQR)**				
All	0.01 (0.01; 0.04)	0.01 (0.01; 0.01)	0.01 (0.01; 0.01)	0.01 (0.01; 0.01)
Premenopause	0.06 (0.01; 0.20)	–	–	–
Perimenopause	0.01 (0.01; 0.07)	0.01 (0.01; 0.02)	0.01 (0.01; 0.01)	^a^
Postmenopause	0.01 (0.01; 0.01)	0.01 (0.01; 0.01)	0.01 (0.01; 0.01)	0.01 (0.01; 0.01)

AMH: Anti-Mullerian hormone; BMI: body mass index; FSH: follicle-stimulating hormone; LH: luteinizing hormone; SHBG: sex hormone binding globulin.

^a^Estimates not calculated for cells with less than 5 individuals.

**Table 2:** Distribution of lifestyle and reproductive risk factors, and educational achievement (N = 1,608).

	N (%)
**BMI at first assessment**	
Normal^a^	805 (50.1)
Overweight	536 (33.4)
Obese	266 (16.5)
**Smoking status**	
Never smoker	803 (54.6)
Former smoker	520 (35.3)
Current smoker	149 (10.1)
**Alcohol intake frequency**	
Never or less than 4 times a month	449 (40.5)
2 to 3 times a week	367 (33.1)
4 or more times a week	293 (26.4)
**Parity**	
1	183 (11.4)
2	534 (33.2)
3	396 (24.6)
4+	495 (30.8)
**Age at menarche**	
Early (≤ 11 years)	240 (16.2)
Average (12–14 years)	1,039 (70.0)
Late (≥ 15 years)	204 (13.8)
**Educational achievement**	
CSE/vocational degree/O-level	637 (42.3)
A-level	475 (31.6)
University degree	393 (26.1)

A-level: advanced level; BMI: body mass index; CSE: certificate of secondary education; O-level: ordinary level.

^a^26 women (1.6%) had BMI < 18.5 kg/m^2^ and were included in the normal BMI category.

### Reproductive hormone patterns of change by reproductive and chronological age

For all four hormones, reproductive age patterns of change were a slightly better fit to the data than chronological age patterns, though the best fit was the model including both reproductive and chronological age (Supplementary Table 3). Regression coefficients for each model are presented in Supplementary Table 4.

**Figure 2:**
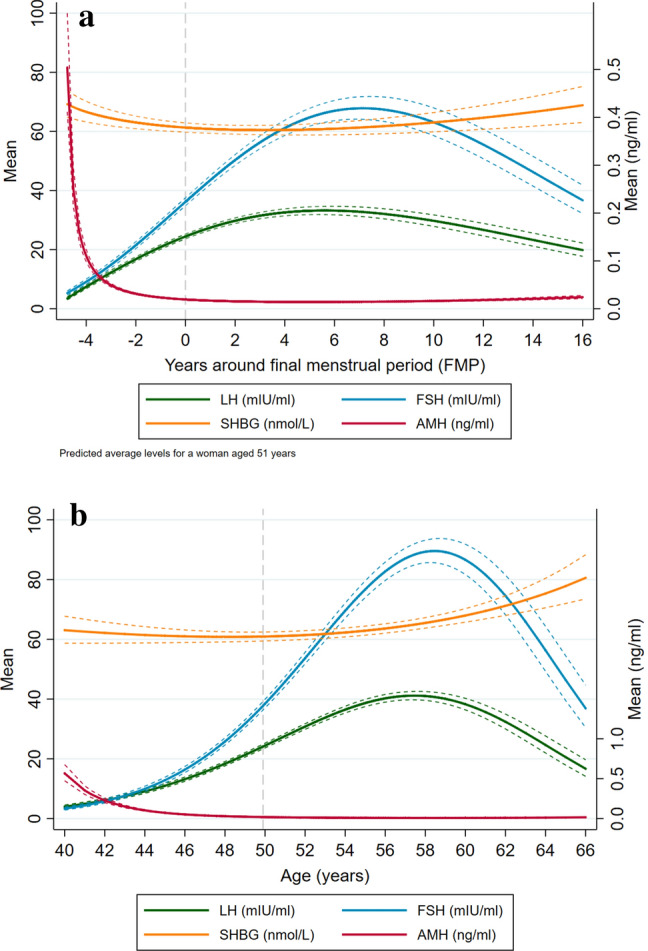
Average predicted population means (95% CI) for reproductive hormones across reproductive age (**A**) and chronological age (**B**). Dashed vertical line corresponds to menopause (**A**) or average age of menopause in the study (**B**).

Figure 2A shows the patterns of change in hormone levels by reproductive age, adjusted for chronological age. These patterns represent average predicted population means derived from multilevel models for a woman aged 51 years. Both LH and FSH increased until ~ 5 and 7 years after the menopause, respectively, after which they decreased (Fig. 2A, Supplementary Table 5). For instance, for a woman aged 51 years who is 2 years before the FMP, the FSH level is about 20 mIU/ml, increasing to 67 mIU/ml 7 years after the FMP and decreasing to 40 mIU/ml 15 years postmenopause. The rate of increase was higher for FSH, which also had a higher rate of decrease in later postmenopause than LH. SHBG decreased slightly until about 4 years postmenopause and started to increase subsequently, but changes in SHBG levels were small. AMH decreased markedly before the menopause and remained low thereafter. Compared to results unadjusted for age (Supplementary Figure 1), the shapes were overall similar, but hormone levels, especially at postmenopause, were generally lower, and FSH increased until ~ 9 years postmenopause, after which it decreased.

Hormone patterns of change by chronological age are shown in Fig. 2B. Both LH and FSH increased with age, with a higher rate of increase from about 46 years until approximately 58 years, after which they started to decline (Fig. 2B, Supplementary Table 6). For instance, FSH levels are, on average, 13 mIU/ml in a woman aged 45 years, 39 mIU/ml in a woman aged 50, 77 mIU/ml in a woman aged 55, 89 mIU/ml in a woman aged 60, and 46 mIU/ml in a woman aged 65 years. The increase in FSH was more accentuated than in LH and so was its decline. SHBG levels changed little until about 55 years, after which they started to increase. AMH declined with age until about 48 years and reached undetectable levels thereafter.

Results for reproductive and chronological age models were similar when data were restricted at the 5th and 95th centiles of FMP and when only women with 3 or 4 repeated measures were analysed (Supplementary Table 3, Supplementary Figures 2 and 3). Results were also similar when analyses were repeated using GEE (Supplementary Figure 4). LH and FSH increased with chronological age in both women excluded and included in the analyses, though with slightly different shapes (Supplementary Figure 5). SHBG increased with ageing in those excluded from the analyses, whilst little change was observed in those included. AMH levels before age 50 were higher in those excluded from the analyses and decreased at later age than those included in the analyses. Women whose age at menopause was unknown were younger than those with known age at menopause, had lower levels of LH and FSH and higher levels of SHBG and AMH, and were more likely to be obese and to have lower frequency of alcohol intake and lower education (Supplementary Table 7). When analysis was performed by menopausal stages (Supplementary Figure 6), results were similar to those presented in our main analyses.

### Associations of lifestyle and reproductive characteristics with reproductive age patterns of change in hormones

**Figure 3:**
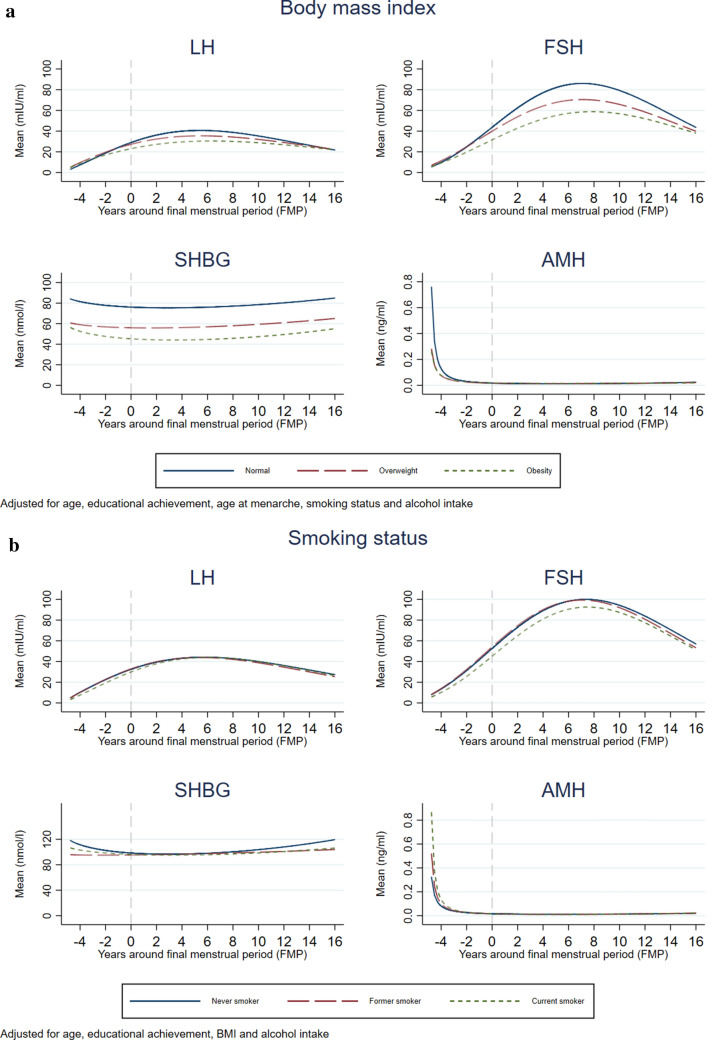

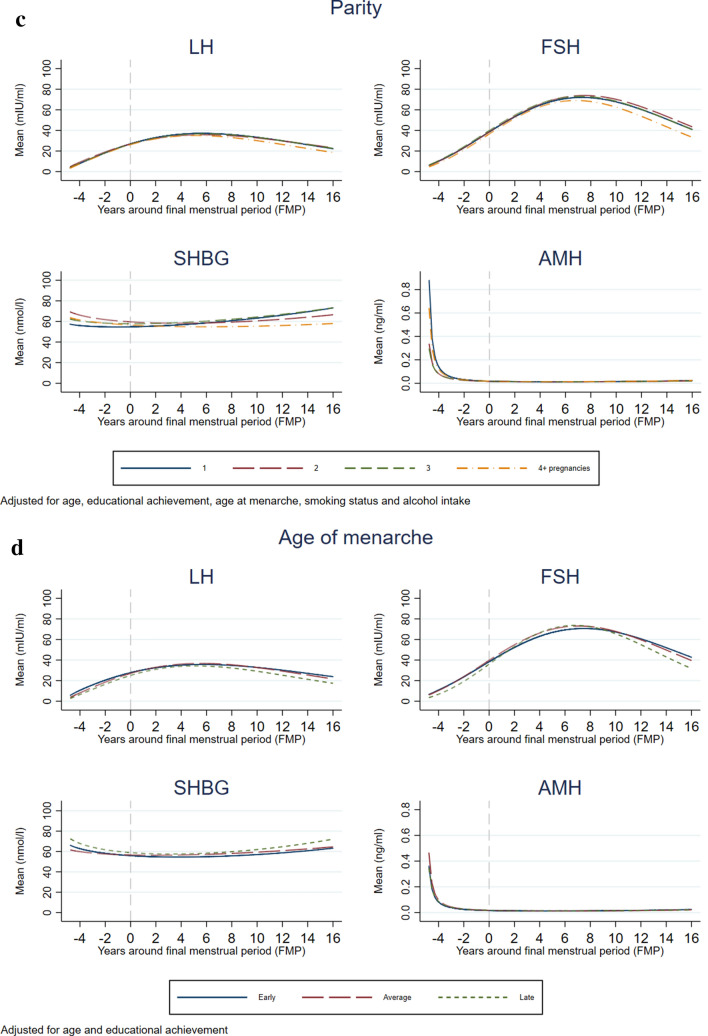
Association of lifestyle and reproductive factors with mean hormone levels at different reproductive ages. In these analyses, all covariates used in the adjustment were set to the mean value or the reference category: age (51 years), education (CSE/Vocational degree/O-level), body mass index (< 25 kg/m^2^), smoking status (never smoker), alcohol intake (never or less than 4 times a month), age at menarche (13 years).

Figures 3A (BMI), 3B (smoking status), 3C (parity) and 3D (age at menarche) show the patterns of change in the four hormones by reproductive age according to the lifestyle and reproductive factors, adjusted for observed confounders. Average predicted values for each hormone are presented in Supplementary Table 8 and p-values for interactions are presented in Supplementary Table 9. Rates of increase of LH and FSH were inversely associated with BMI, with the lowest increase observed in obese women. For example, FSH increased on average 79% from the menopause to 5 years postmenopause in women with normal weight, whilst this increase was on average 58% in those with obesity. Patterns of change in SHBG with reproductive age were similar in all three BMI categories, with levels consistently lowest in obese women, highest in those who were normal weight and in between in women who were overweight. Obese and overweight women had lower rates of decrease in AMH compared to women with normal BMI. Patterns of change in LH and FSH with reproductive age were similar across all smoking categories, with FSH levels consistently lowest in current smokers. Current and former smokers had lower rates of changes in SHBG than never smokers, as well as lower levels throughout reproductive age. Rates of decrease in AMH were higher in current smokers, followed by former smokers, compared to women who never smoked, and AMH levels before menopause followed the same pattern. Patterns of change in the reproductive hormones did not differ by alcohol intake, but women who had a higher frequency of alcohol intake had higher levels of FSH from 2 years after the menopause, lower levels of SHBG throughout reproductive age, and higher levels of AMH before the menopause than those with lower alcohol intake frequency (Supplementary Figure 7, Supplementary Table 8).

Patterns of change in LH and FSH with reproductive age did not differ by parity, but women who had 4 or more pregnancies had lower levels of these hormones from 6 years postmenopause. There was an association between parity and SHBG patterns of change, such that in women with more than 1 pregnancy there was a sharper decrease before the FMP and then a plateau or slight increase after the FMP in comparison to women who had experienced only one pregnancy, in whom there was little decrease before the menopause and a steeper increase after. There were some differences in AMH decline and levels prior to the FMP, with slower rate of decline and lower mean levels in women with 2–3 pregnancies than women with 1 or 4 or more pregnancies. Patterns of change in reproductive hormones did not notably differ by age of menarche, but levels of LH and FSH were lower before the menopause and from about 8–10 years postmenopause in women with late menarche compared to women with average age at menarche.

Results were similar when analyses were performed only in women with complete information on risk factors and confounders (Supplementary Table 9, Supplementary Figure 8A–E).

### Associations of age at menopause with chronological age patterns of change in hormones

**Figure 4:**
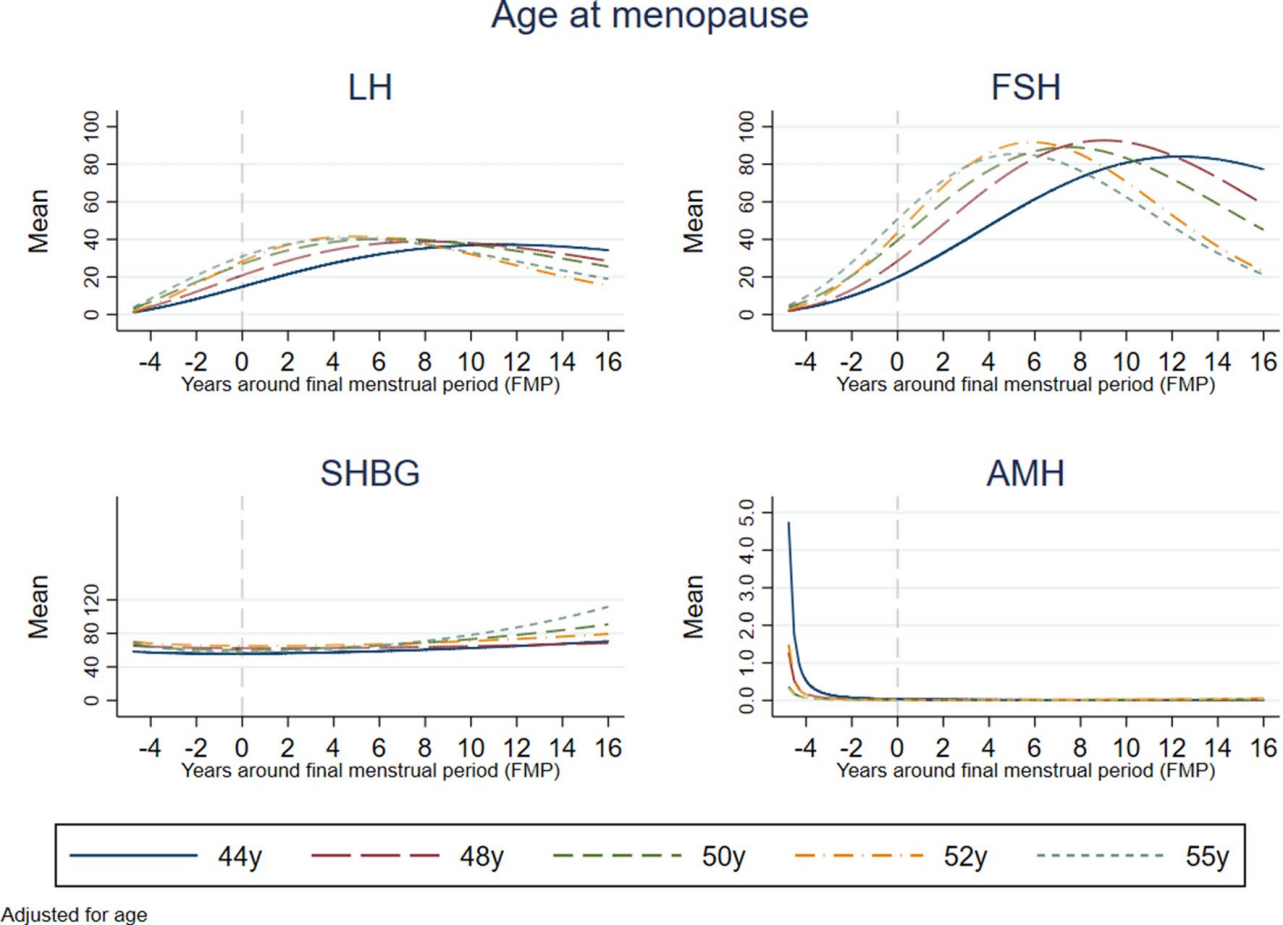
Association of age at menopause with mean hormone levels by reproductive age. Quintiles of age at menopause [mean age (SD)]: Q1: 44.2 (2.9), Q2: 48.4 (0.6), Q3: 50.1 (0.5), Q4: 52.2 (0.7), Q5: 54.8 (1.5).

Figure 4 shows the patterns of change in the four hormones by reproductive age according to the age at menopause (in quintiles). Women with earlier age of menopause had lower levels of LH and FSH until about 6 years postmenopause and higher levels thereafter, and the decline in these hormones was later than in those with average age of menopause (50 years). Those with later age at menopause had lower postmenopausal levels of LH and FSH and these hormones started to decline earlier than in those with average age of menopause. Women who experienced the menopause at later age had higher increase in SHBG from about 8 years after the FMP, and AMH levels and decline were lower than observed for those with average age of menopause. Those with earlier age at menopause had the highest levels of AMH about 4 years before the FMP, and the decline in this hormone was slightly later than in those with menopause at on average 50 years.

## Discussion

We have shown that both reproductive and chronological age are associated with patterns of change in LH, FSH, SHBG and AMH in mid-life in women and that BMI, smoking and parity are associated with the patterns of one or more of these hormones. These findings, if replicated, suggest that any exploration of the role of change in reproductive hormones around the time of the menopause with later adverse health outcomes must control for both reproductive and chronological age, and consider adjusting for BMI, smoking and parity (depending on the likelihood of these factors influencing the outcome of interest).

Consistent with our findings, previous studies with longitudinal data have shown that levels of LH and FSH increase with reproductive and chronological age at least up to the early postmenopause, and that postmenopausal levels of these hormones remain higher than premenopausal levels^8–10,25,37^. However, across our and other studies, there is variation in whether, after a period of increase, levels then appear to remain stable^8–10^ or decline^13,38^; if there is a decline, the age at which this occurs seems to vary. Three previous studies reported that the increase in FSH stabilised after 2 years postmenopause^8–10^; two of those studies included fewer than 650 women and the largest one had 1,215 women, and all had more repeat measures than our study (varying from average 5.3 to 9.1 measures per women). An additional two studies (a Swedish study of 160 women with an average of 16 repeat measures^13^, and a US study of 856 women with up to 12 follow-up visits)^38^ showed postmenopausal declines in LH and FSH, as in our study, with these declines beginning earlier, around 1–4 years postmenopause. The differences between studies in stabilise/decline and when the decline occurs is likely to be due to differences in length of follow-up, number of repeats, potential methodological differences relating to assays, and possibly distribution of other characteristics, such as BMI, smoking and parity. The decline in both LH and FSH might be due to pituitary desensitization in the positive feedback of oestrogen, with a relative hypothalamic-pituitary insensitivity to oestrogen in ageing women^39^. Increased adiposity might also play a role, as observed in this and other studies^26,28^. Our study has the longest postmenopausal follow-up to date, and the mechanisms that explain reproductive hormone changes in a longer postmenopausal period are not known and require further exploration.

Although changes in SHBG were small, we observed a slight decrease up to 4 years postmenopause, followed by an increase small in magnitude. Differently from our study, three of the four longitudinal studies that have assessed patterns of change in SHBG reported decreases after the menopause, with no increase thereafter^11,13,40^. One showed a marked increase (of 81%) across the perimenopause^14^. Those studies were smaller (all including fewer than 450 women) and had shorter follow-up periods (up to 8 years postmenopause). Our findings corroborate results from cross-sectional studies to some extent, in which SHBG has been shown to be inversely associated with age between 20 and 60 years, after which it is higher at older ages^21,41,42^. The marked decline, to undetectable levels, in AMH before the menopause has been consistently shown in longitudinal studies^15,16,22,43^.

The 2011 STRAW + 10 updated previous reproductive categories based on a detailed review of evidence and expert discussion, with a particular focus on evidence of the critical changes in hypothalamic-pituitary-ovarian function that occur before and after the FMP^33^. LH and FSH are central to this axis, with their secretion from the pituitary and circulating levels controlled by feedback loops involving gonadotropin-releasing hormone from the hypothalamus and, in women, oestrogen and progesterone levels from the ovaries. The workshop concluded that “although smoking and BMI influence [reproductive] hormonal levels and the timing of [menopausal] transition, these factors do not alter the trajectory of change in bleeding patterns or hormonal levels with reproductive aging. Therefore, the STRAW + 10 staging system is applicable to women regardless of age, demographic, BMI, or lifestyle characteristics”^13^. However, our results and those from other longitudinal studies (summarised in Supplementary Table 1) challenge these conclusions, showing that lifestyle and reproductive factors are not only associated with hormone levels but also with different patterns of reproductive age-related changes. In light of major changes in these risk factors over the last decades, such as decrease in smoking prevalence in mid-aged women in high-income countries and increase in overweight/obesity^44^, it may be important to take these into consideration when defining reproductive categories based on STRAW criteria. If our results are replicated, future versions of these categories should also consider whether category definitions should vary between women with differing levels of BMI, smoking and parity. Although differences in hormone changes observed by parity categories could be explained by age at pregnancy, post hoc analysis including age at first pregnancy in the adjustment did not change the results (Supplementary Figure 9).

### Strengths and limitations

This is the largest prospective study to date with measures of four reproductive hormones that span the late reproductive period and postmenopause. The average 5-year follow-up period with up to four repeat measures in women of different baseline ages allowed the description of hormone patterns of change from 4 years before to 16 years after the menopause, a longer postmenopausal period than described in previous studies. We were also able to explore the influence of lifestyle and reproductive factors on the hormonal patterns of change by reproductive ageing, adjusting for observed confounders, which very few previous longitudinal studies have done.

We used multilevel models, which allow all women with at least one measure of the hormone levels to be included in the analysis under the MAR assumption, i.e. associations do not differ in those who have fewer repeat measures. It is not possible to test this assumption directly. Sensitivity analysis of women who had 3 or 4 repeat measures showed similar results to those with at least one repeat measure, and similar results were also observed using GEE, providing support that this assumption is not notably violated as we would expect results to be different if data were MCAR.

We restricted the follow-up in the second, third and fourth clinic assessments to women who were pre- or perimenopausal and aged 40 years or older at the first assessment, so that they were likely to transition through the menopause. This may have introduced some selection bias. However, given the focus of this paper is on changes in hormones across the menopausal transition, had these women been included, they were unlikely to have contributed to the results presented here. The mean reproductive age of this excluded group would have moved closer to their future FMP over the three assessments, however, due to their young age, most are unlikely to have started to experience menopause-related changes in their sex hormones. Furthermore, the selection by age means that bias is unlikely because age, the source of selection (missingness), is included in all of our analysis models, which means that the MAR assumption of multilevel models is unlikely to be violated.

We used fractional polynomials to model the non-linear associations; although these can be influenced by outliers, analyses excluding women in the bottom and top 5% of the distribution of FMP showed similar results to the analysis including all women. We compared models of hormone patterns by reproductive and chronological age (separately and combined) and showed that models with both reproductive and chronological age were the best fit of our hormonal data, suggesting the importance of the menopause transition in the patterns of these hormones. We only included women who are known to have gone through a natural menopause, and excluded those whose age at menopause was unknown. This could have introduced selection bias, which might be reflected by the lower average age of menopause than found in other studies^45^. However, the mean age of menopause was similar to that observed in a meta-analysis of 17 studies in seven high-income countries, including the UK (mean age 50.2, SD 4.4)^46^. We performed sensitivity analysis including women whose age at menopause was unknown and the results, though slightly different than the main analysis, followed what it would be expected given the younger age of those with unknown menopause status compared to those with known age at menopause. Therefore, it is unlikely that selection bias have substantially influenced our results.

We did not have data on oestradiol. Previous studies show (as expected biologically) that oestradiol mirrors FSH, such that as FSH increases between 2-years before and 2-years after the FMP, oestradiol decreases, with the two subsequently stabilizing at high and low levels, respectively^9,10,12,13^. Our study is predominantly of White European origin women, and previous studies have shown ethnic differences in reproductive hormone levels^18,26,28^. Whilst ethnic differences in mean levels do not mean that patterns of change with reproductive and chronological age, or associations of lifestyle and reproductive factors with these patterns, will differ by ethnicity, it is possible that our findings might not generalise to women of other race/ethnic groups. As our study recruited women during an index pregnancy and only followed those with a live birth from that pregnancy, all participants had at least one live birth and we cannot assume that our findings would generalise to women with no previous pregnancies or live births. In particular, the association of parity with changes in reproductive hormones may differ in studies that also include nulliparous women. As women without an observed natural menopause were excluded, our results might not generalise to all women. However, the results for patterns of change in hormones by chronological age were broadly similar in the whole sample of women compared to when restricted to women who had gone through the menopause. The modest differences in patterns of change, mostly seen with SHBG, may be related to differences in ages between those included in our main analyses and the whole cohort, with those who have gone through the menopause being older on average than the whole cohort.

## Conclusions

Our results add to the relatively sparse literature of studies with repeat measurements of reproductive hormones (see Supplementary Table 1) by exploring the relation of both chronological and reproductive age, and of lifestyle and reproductive factors with hormone levels. Our, and previous studies, show increases in LH and FSH with reproductive age, with levels remaining higher in postmenopause compared to premenopause, and that AMH declines markedly just before the menopause to undetectable levels. Novel findings from our study are the importance of both reproductive and chronological age to the reproductive hormone patterns of change, and the associations of BMI, smoking and parity with these patterns. Future studies of the associations of these hormones with health outcomes should consider potential confounding by chronological and reproductive age, as well as BMI, smoking and parity, and the current STRAW criteria for reproductive stage categories, which assume that lifestyle and reproductive factors do not influence hormone patterns, may need to be reconsidered.

## Supplementary information

Supplementary Information.
