# Supplementary material for: Longitudinal changes in reproductive hormones through the menopause transition in the Avon Longitudinal Study of Parents and Children (ALSPAC)
Source: Sci Rep. 2020 Dec 4;10:21258. doi: 10.1038/s41598-020-77871-9 (PMC7718240; doi:10.1038/s41598-020-77871-9)
Supplement: Supplementary file 1 — Supplementary Information. [file 41598_2020_77871_MOESM1_ESM.pdf]

Longitudinal changes in reproductive hormones through the menopause transition in the Avon Longitudinal Study of Parents and Children (ALSPAC)

Supplementary material

Ana Goncalves Soares*[1,2], Fanny Kilpi[1,2], Abigail Fraser[1,2,3], Scott M Nelson[3,4], Naveed Sattar[5], Paul I Welsh[5], Kate Tilling[§1,2,3], Deborah A Lawlor[§1,2,3]

Affiliations:

[1] MRC Integrative Epidemiology Unit at the University of Bristol, Bristol, UK

[2] Population Health Science, Bristol Medical School, University of Bristol, Bristol, UK

[3] Bristol NIHR Biomedical Research Centre, Bristol, UK

[4] School of Medicine, Dentistry and Nursing, University of Glasgow, Glasgow, UK

[5] Institute of Cardiovascular and Medical Sciences, University of Glasgow, Glasgow, UK

[§] contributed equally as senior authors

Supplementary methods

Weight and height were measured in all clinics according to standard protocols, with women wearing light clothes and unshod. Weight was measured using an electronic scale with accuracy of 100g (Tanita TBF-401) and height was measured using a Harpenden stadiometer and recorded to the nearest 1mm.

We used directed acyclic graphs and expert knowledge to decide what to include as potential confounders for the associations of lifestyle and reproductive factors with reproductive age-related trajectories of reproductive hormones. Confounders were defined as factors that were known or could plausibly influence both the putative risk factor and reproductive hormones. Confounders for each of the risk factors were:

1. For body mass index (BMI): chronological age (in years), educational achievement (categories: CSE/ vocational degree/ O-level, A-level, university degree), age of menarche (in years), smoking status (categories: never, former and current smoker) and alcohol intake (categories: never or less than 4 times/month, 2-3x/week, 4 or more times/week).
2. For smoking status (units and categories as for BMI): chronological age, educational achievement, BMI and alcohol intake.
3. Alcohol intake (units and categories as for BMI): chronological age, educational achievement, BMI and smoking status.
4. For parity (units and categories as for BMI): chronological age, educational achievement, age at menarche (in years), smoking status and alcohol intake. Post hoc analysis was performed further including age at first pregnancy as a confounder.
5. For age of menarche (units and categories as for BMI): chronological age and educational achievement.

Continuous confounders were centred at the average value for interpretation purposes; age was centered at 51 years and age at menarche at 13 years.

Fractional polynomials

The best-fitting function was selected from a family of flexible polynomial functions. In brief, with simple (single-level) linear regression, the model deviance of each of eight powers (–2, –1, –0.5, 0, 0.5, 1, 2, 3) is used to identify the best-fitting single polynomial. All possible combinations of pairs of these polynomials are then examined, and again the model deviance is used to select the best-fitting model containing two powers. The difference between the model deviance for the best-fitting polynomials of degrees 1 and 2 are then compared to the χ^2 distribution with 2 degrees of freedom to test whether the addition of an extra polynomial

term significantly improves the model (Royston & Altman, 1994). In the multilevel framework, the method is similar, and it has been described in detail elsewhere (Tilling, 2014). The difference in model deviance between the best-fitting polynomial of degree 2 and that of degree 1 is compared to the $\chi2$ distribution with 5 degrees of freedom, since one extra power, one fixed coefficient and three random parameters are being estimated. When using fractional polynomials, time/age must be strictly greater than zero, and a constant can be added to achieve this strict positivity. In our models for reproductive age, we added a constant of 5, given the lowest time since the final menstrual period was -4.7 years.

References

- Tilling K, Macdonald-Wallis C, Lawlor DA, Hughes RA, Howe LD. Modelling childhood growth using fractional polynomials and linear splines. Ann Nutr Metab. 2014;65(2-3):129-38. doi:10.1159/000362695
- Royston P, Altman DG. Regression Using Fractional Polynomials of Continuous Covariates: Parsimonious Parametric Modelling. J R Stat Soc C. 1994; 43(3): 429-467. doi:10.2307/2986270.

Supplementary Tables and Figures

Supplementary Table 1. Summary of longitudinal studies that assessed the association of lifestyle/ reproductive factors with changes in follicle-stimulating hormone (FSH), luteinizing hormone (LH), sex-hormone binding globulin (SHBG) and anti-Müllerian hormone (AMH) by reproductive and/or chronological age

Author, year Country	Sample characteristics	Outcomes	Repeat measures	Lifestyle/ reproductive factors	Covariates	Results
Gohari, 2016 [16]<br><br>Iran	266 women aged 20-50 years at baseline (regular and predictable menstrual cycles)<br><br>Tehran Lipid and Glucose Study (TLGS)	Change of AMH with chronological age (20-50 years)	Up to 3 repeat measures over a 6.5-year follow-up	BMI	None	AMH decreased with chronological age, and the rate of change was the fastest in the oldest group (40-50 years) and<br><br>BMI was not associated with AMH.
Tepper, 2012 [28]<br><br>United States	1,316 women with natural menopause aged 42-52 y at the baseline<br><br>Study of Women's Health Across the Nation (SWAN)	Change of oestradiol and FSH with reproductive age (8 years before to 8 years after the menopause)	12,586 observations over an average 9.5-year follow-up	BMI, race/ethnicity, physical activity, smoking, health status, difficulty in paying basics, education and site	Age and cycle day of blood draw	Four trajectories of oestradiol were identified: slow decline (26%), flat (28.6%), rise and then gradual decline (13.1%) and rise followed by steep decline (31.5%).<br><br>Only race/ethnicity and BMI were associated with oestradiol trajectories. Women with slow decline trajectory were more likely to be Chinese or Japanese; women with flat trajectory were more likely to be African American, overweight or obese, and women with rise/slow decline were more likely to be Chinese or overweight.<br><br>Three distinct trajectories of FSH were found: low (10.6%), medium (48.7%) and high (40.7%) rising trajectories. Race/ethnicity and BMI were related to FSH trajectory groups, but not smoking, physical activity, or demographic variables.

						Women following low trajectory were more likely to be African American, Hispanic, overweight or obese. Women following the medium trajectory were more likely to be Japanese and overweight/obese.
Randolph, 2011 [9]<br><br>United States	1,215 women with natural menopause (median age 51.5y)<br><br>Study of Women's Health Across the Nation (SWAN)	Change of oestradiol and FSH with reproductive age (8 years before to 8 years after the menopause)	9,435 observations over a 9-year follow-up	Obesity, smoking and race/ethnicity	None	The patterns of change in FSH and oestradiol in relation to FMP were not different when comparing obese and nonobese women, although different mean levels were observed. FSH and oestradiol changes were less pronounced in obese than in non-obese women, especially after the FMP.<br><br>Smokers and non-smokers had overlapping trajectories of mean FSH across reproductive age. The mean trajectory of oestradiol was higher among smokers than non-smokers.<br><br>Change in FSH was not associated with race/ethnicity. Decline in oestradiol before the FMP started first in African American (2.0 years before the FMP) and Caucasian (1.9 y) women, followed by Japanese (1.6 y) and Chinese (1.3y) women. Oestradiol stabilised later in Japanese women (1.7y after the FMP) than other race/ethnic groups (1.9-2.0y after the FMP).
Sowers, 2010 [27]<br><br>United States	50 women, aged 41 (SD 2.6) at baseline	Change of AMH, inhibin B, and FSH with reproductive (10 years before and 10 years after the	6 yearly measures of women who were in their pre- and	Smoking, obesity and insulin resistance (HOMA-IR)	None	Women who smoked had steeper slope of their AMH levels in relation to age at FMP (AMH declined faster in smokers). There was no interaction

	Sub-sample of Michigan Bone Health and Metabolism Study cohort	menopause) and chronological age (28 to 60 years)	early menopause stages.			between AMH and obesity or HOMA-IR in relation to time to FMP.<br><br>There was no interaction with smoking, obesity or HOMA-IR and both inhibin B and FSH in relation to time to FMP.
Freeman, 2010 [25]<br><br>United States	436 women aged 35-47 years at baseline<br><br>Penn Ovarian Aging Study	Change in oestradiol, FSH and inhibin B with reproductive age (premenopausal, late premenopausal, early transition, late transition and postmenopausal)	3,578 observations over a 12-year follow-up	Race/ethnicity, adiposity (BMI, WC and WHR)	Race, age and smoking	There was no interaction of race/ethnicity with oestradiol, FSH or inhibin B.<br><br>Associations of BMI with the hormones differed by menopausal stage.<br><br>Inhibin levels were lower in obese and overweight women at the premenopausal stages and switched in the late transition stage, with the normal BMI group having the lowest levels and the obese group the highest levels.<br><br>Associations of BMI with FSH differed in postmenopause; obese women had the lowest FSH levels and normal-weight women had the highest FSH levels.<br><br>Inhibin B levels were lower in obese and overweight women at the premenopausal stages and switched in the late transition stage, with the normal BMI group having the lowest levels and the obese group the highest levels.<br><br>Obesity measured by WC and WHR followed similar patterns.

Sowers, 2008 [8]<br><br>United States	629 women aged 24–44 y at baseline<br><br>Michigan Bone Health and Metabolism Study cohort	Change in FSH with reproductive (10 years before and 10 years after the menopause) and chronological age (28 to 60 years)	5,757 observations over a 14-year follow-up	Smoking, parity and age at menarche	Baseline age and baseline FSH.	Smoking, parity and age at menarche were not associated with FSH change.
Randolph, 2004 [26]<br><br>United States	3,527 women aged 42–52 y at the baseline<br><br>Study of Women's Health Across the Nation (SWAN)	Change in oestradiol and FSH with chronological age (42 to 54 years)	7,648 measures over 3 consecutive annual visits	BMI, smoking, diabetes, parity, alcohol intake and socioeconomic status	None	Increasing BMI was associated with decreasing concentrations of oestradiol and FSH. BMI was inversely associated with oestradiol in premenopausal women; among late perimenopausal and postmenopausal women, increasing BMI was associated with increasing concentrations of oestradiol.<br>The effect of BMI on decreased FSH became more pronounced as women transitioned through menopause.<br><br>Current smoking was positively associated with FSH concentrations, and diabetes was negatively associated with FSH levels.<br><br>Parity, socioeconomic status, alcohol intake and physical activity were not associated with either oestradiol or FSH.

AMH: Anti-Mullerian hormone; BMI: body mass index; FMP: final menstrual period; FSH: follicle-stimulating hormone; LH: luteinizing hormone; SHBG: sex hormone binding globulin; WC: waist circumference; WHR: waist-hip ratio

Supplementary Table 2. Characteristics of the data before and after imputation

	% missing	Before imputation %	After imputation %
BMI	0.6%	n = 1,607	n = 1,608
Normal		50.1	50.0
Overweight		33.4	33.4
Obese		16.5	16.5
Smoking status	8.5%	n = 1,472	n = 1,608
Never smoker		54.6	53.9
Former smoker		35.3	35.7
Current smoker		10.1	10.4
Alcohol intake frequency	31.0%	n = 1,109	n = 1,608
Never or less than 4x/ month		40.5	40.7
2 to 3 times a week		33.1	32.8
4 or more times a week		26.4	26.5
Age at menarche		n = 1,483	n = 1,608
Early (≤ 11 years)	7.8%	16.2	16.7
Average (12-14 years)		70.0	69.8
Late (≥ 15 years)		13.8	13.5
Educational achievement	6.4%	n = 1,505	n = 1,608
CSE / Vocational degree/ O-level		42.3	42.7
A-level		31.6	31.5
University degree		26.1	25.8

A-level: advanced level; BMI: body mass index; CSE: certificate of secondary education; O-level: ordinary level;

Supplementary Table 3. Characteristics of the model fit for each reproductive hormone according to reproductive and chronological age.

			Reproductive age				Chronological age			
	**N participants**	**N measures**	**Powers**	**df**	**AIC**	**BIC**	**Powers**	**df**	**AIC**	**BIC**
**Main analysis**										
Unadjusted										
LH	1,608	4,037	0.5, 1	5	7606	7638	3, 3	5	7805	7836
FSH	1,608	4,037	1; 1	5	8562	8594	3, 3	5	8938	8969
SHBG	1,608	4,037	1; 1	5	2929	2960	3, 3	5	2934	2966
AMH	1,608	4,037	0.5; 1	5	8846	8877	1, 1	5	8860	8892
Adjusted for chronological age										
LH	1,608	4,037	0.5, 1	6	7495	7533				
FSH	1,608	4,037	1, 1	6	8401	8438				
SHBG	1,608	4,037	1; 1	6	2922	2960				
AMH	1,608	4,037	0.5; 0.5	6	8580	8618				
**Sensitivity analysis: restricted at the 5th and 95th centiles of FMP**										
Unadjusted										
LH	1,552	3,640	0.5, 0.5	5	6517	6548	2, 3	5	6644	6675
FSH	1,552	3,640	0.5, 1	5	7483	7514	3, 3	5	7732	7763
SHBG	1,552	3,640	0.5, 2	5	2640	2671	3, 3	5	2650	2681
AMH	1,552	3,640	0.5, 0.5	5	7557	7588	0.5, 1	5	7291	7322
Adjusted for chronological age										
LH	1,552	3,640	0.5, 0.5	6	6405	6442				
FSH	1,552	3,640	0.5, 1	6	7313	7350				
SHBG	1,552	3,640	1, 2	6	2638	2675				
AMH	1,552	3,640	0, .0.5	6	7283	7320				
**Sensitivity analysis: only women with 3 or 4 repeat measures**										
Unadjusted										
LH	818	2,862	0.5, 1	5	4656	4686	2, 3	5	5017	5047
FSH	818	2,862	0.5; 1	5	5443	5473	3, 3	5	5992	6022
SHBG	818	2,862	1; 1	5	1521	1551	3, 3	5	1539	1569
AMH	818	2,862	0.5; 0.5	5	5230	5259	0.5, 1	5	5780	5810
Adjusted for chronological age										
LH	818	2,862	0.5, 1	6	4643	4678				
FSH	818	2,862	0.5, 1	6	5418	5454				
SHBG	818	2,862	1; 1	6	1520	1556				
AMH	818	2,862	0.5; 0.5	6	5188	5223				

AIC: Akaike Information Criterion; AMH: Anti-Mullerian hormone; BIC: Bayesian Information Criterion; df: degrees of freedom; FMP: final menstrual period; FSH: follicle-stimulating hormone; LH: luteinizing hormone; SHBG: sex hormone binding globulin

Supplementary Table 4. Regression coefficients for the fractional polynomial models

Hormone	Model	Parameter	Estimate	SE	p-value
LH	1	Intercept	0.02	0.10	0.8492
		FP term 1	2.07	0.07	<0.001
		FP term 2	-0.29	0.01	<0.001
	2	Intercept	0.49	0.11	<0.001
		FP term 1	1.91	0.07	<0.001
		FP term 2	-0.29	0.01	<0.001
	3	Intercept	-4.15	0.28	<0.001
		FP term 1	5.46e-04	2.38e-05	<0.001
		FP term 2	1.24e-04	5.51e-06	<0.001
FSH	1	Intercept	0.99	0.07	<0.001
		FP term 1	0.92	0.02	<0.001
		FP term 2	-0.25	0.01	<0.001
	2	Intercept	1.54	0.08	<0.001
		FP term 1	0.81	0.03	<0.001
		FP term 2	-0.23	0.01	<0.001
	3	Intercept	-5.96	0.33	<0.001
		FP term 1	6.91e-04	2.75e-05	<0.001
		FP term 2	1.57e-04	6.36e-06	<0.001
SHBG	1	Intercept	4.20	0.03	<0.001
		FP term 1	-0.04	0.01	<0.001
		FP term 2	0.02	3.57e-03	<0.001
	2	Intercept	4.28	0.04	<0.001
		FP term 1	-0.06	0.01	<0.001
		FP term 2	0.02	3.62e-03	<0.001
	3	Intercept	4.44	0.15	<0.001
		FP term 1	-3.73e-05	1.27e-05	0.0033
		FP term 2	8.86e-06	2.93e-06	0.0025
AMH	1	Intercept	2.71	0.19	<0.001
		FP term 1	-4.50	0.15	<0.001
		FP term 2	0.96	0.04	<0.001
	2	Intercept	1.93	0.20	<0.001
		FP term 1	-4.34	0.15	<0.001
		FP term 2	1.00	0.04	<0.001
	3	Intercept	71.22	2.72	<0.001
		FP term 1	-6.61	0.25	<0.001
		FP term 2	1.31	0.05	<0.001

Model 1: reproductive age; Model 2: reproductive age adjusted for chronological age; Model 3: chronological age
AMH: Anti-Mullerian hormone; FP: fractional polynomial; FSH: follicle-stimulating hormone; LH: luteinizing hormone; SHBG: sex hormone binding globulin

Supplementary Table 5. Average predicted means for reproductive hormones across reproductive age.

Years since the FMP	LH (mIU/ml) Mean (95% CI)	FSH (mIU/ml) Mean (95% CI)	SHBG (nmol/ml) Mean (95% CI)	AMH (ng/ml) Mean (95% CI)
-5.0 to -4.0	6.55 (5.91; 7.26)	8.20 (7.40; 9.09)	67.14 (63.53; 70.94)	0.15 (0.13; 0.18)
-3.9 to -3.0	9.96 (9.23; 10.75)	12.02 (11.07; 13.05)	65.41 (62.45; 68.52)	0.07 (0.06; 0.08)
-2.9 to -2.0	14.92 (14.15; 15.73)	18.68 (17.58; 19.85)	63.58 (61.29; 65.96)	0.04 (0.04; 0.04)
-1.9 to -1.0	18.62 (17.85; 19.42)	24.65 (23.47; 25.88)	62.54 (60.61; 64.54)	0.03 (0.03; 0.03)
-0.9 to 0	22.26 (21.49; 23.05)	31.49 (30.24; 32.79)	61.71 (60.04; 63.43)	0.02 (0.02; 0.02)
0 to 0.9	25.40 (24.60; 26.23)	38.43 (37.07; 39.84)	61.13 (59.61; 62.70)	0.02 (0.02; 0.02)
1.0 to 1.9	28.77 (27.88; 29.69)	47.29 (45.65; 48.99)	60.65 (59.19; 62.15)	0.02 (0.02; 0.02)
2.0 to 2.9	30.65 (29.66; 31.67)	53.26 (51.31; 55.27)	60.48 (58.98; 62.02)	0.02 (0.01; 0.02)
3.0 to 3.9	32.03 (30.93; 33.17)	58.54 (56.23; 60.95)	60.44 (58.84; 62.09)	0.01 (0.01; 0.02)
4.0 to 4.9	32.94 (31.70; 34.22)	63.18 (60.43; 66.06)	60.55 (58.78; 62.36)	0.01 (0.01; 0.01)
5.0 to 5.9	33.22 (31.86; 34.63)	65.96 (62.82; 69.25)	60.76 (58.82; 62.77)	0.01 (0.01; 0.01)
6.0 to 6.9	33.03 (31.55; 34.59)	67.53 (64.00; 71.26)	61.11 (58.93; 63.37)	0.01 (0.01; 0.02)
7.0 to 7.9	32.45 (30.85; 34.14)	67.68 (63.81; 71.78)	61.55 (59.12; 64.08)	0.01 (0.01; 0.02)
8.0 to 8.9	31.53 (29.81; 33.35)	66.56 (62.39; 71.01)	62.09 (59.37; 64.93)	0.02 (0.01; 0.02)
9.0 to 9.9	30.32 (28.49; 32.26)	64.29 (59.87; 69.05)	62.74 (59.70; 65.93)	0.02 (0.01; 0.02)
10.0 to 10.9	28.99 (27.07; 31.04)	61.33 (56.72; 66.30)	63.42 (60.05; 66.97)	0.02 (0.02; 0.02)
11.0 to 11.9	27.51 (25.52; 29.66)	57.66 (52.93; 62.81)	64.19 (60.47; 68.15)	0.02 (0.02; 0.02)
12.0 to 12.9	25.85 (23.79; 28.08)	53.27 (48.48; 58.54)	65.09 (60.95; 69.51)	0.02 (0.02; 0.02)
13.0 to 13.9	24.09 (22.00; 26.39)	48.49 (43.70; 53.79)	66.09 (61.50; 71.03)	0.02 (0.02; 0.02)
14.0 to 14.9	22.51 (20.40; 24.85)	44.09 (39.38; 49.37)	67.06 (62.03; 72.49)	0.02 (0.02; 0.02)
15.0 to 15.9	20.96 (18.84; 23.32)	39.75 (35.16; 44.95)	68.08 (62.60; 74.04)	0.02 (0.02; 0.03)

Predictions are made for a woman aged 51 years.

AMH: Anti-Mullerian hormone; FMP: final menstrual period; FSH: follicle-stimulating hormone; LH: luteinizing hormone; SHBG: sex hormone binding globulin

Supplementary Table 6. Average predicted means for reproductive hormones across chronological age.

Age (years)	LH (mIU/ml) Mean (95% CI)	FSH (mIU/ml) Mean (95% CI)	SHBG (nmol/ml) Mean (95% CI)	AMH (ng/ml) Mean (95% CI)
40 to 40.9	4.00 (3.52; 4.55)	3.33 (2.88; 3.86)	63.05 (58.70; 67.73)	0.57 (0.48; 0.68)
41 to 41.9	5.20 (4.66; 5.80)	4.71 (4.15; 5.35)	62.49 (58.70; 66.52)	0.33 (0.28; 0.38)
42 to 42.9	6.16 (5.58; 6.80)	5.89 (5.26; 6.61)	62.13 (58.71; 65.76)	0.22 (0.20; 0.26)
43 to 43.9	7.63 (7.00; 8.30)	7.84 (7.11; 8.66)	61.75 (58.74; 64.90)	0.15 (0.13; 0.16)
44 to 44.9	9.23 (8.58; 9.94)	10.13 (9.31; 11.03)	61.44 (58.79; 64.20)	0.10 (0.09; 0.11)
45 to 45.9	11.25 (10.57; 11.97)	13.22 (12.30; 14.21)	61.17 (58.86; 63.58)	0.07 (0.07; 0.08)
46 to 46.9	13.44 (12.74; 14.17)	16.82 (15.82; 17.89)	60.98 (58.94; 63.10)	0.05 (0.05; 0.05)
47 to 47.9	16.04 (15.34; 16.78)	21.43 (20.34; 22.58)	60.86 (59.04; 62.74)	0.04 (0.04; 0.04)
48 to 48.9	18.71 (17.99; 19.45)	26.48 (25.30; 27.71)	60.82 (59.16; 62.52)	0.03 (0.03; 0.03)
49 to 49.9	21.68 (20.94; 22.44)	32.50 (31.21; 33.84)	60.85 (59.30; 62.44)	0.02 (0.02; 0.02)
50 to 50.9	24.82 (24.03; 25.63)	39.32 (37.87; 40.83)	60.96 (59.48; 62.48)	0.02 (0.02; 0.02)
51 to 51.9	27.95 (27.10; 28.83)	46.62 (44.96; 48.34)	61.16 (59.71; 62.65)	0.02 (0.02; 0.02)
52 to 52.9	31.18 (30.24; 32.16)	54.72 (52.79; 56.72)	61.46 (60.01; 62.95)	0.01 (0.01; 0.01)
53 to 53.9	34.10 (33.07; 35.17)	62.62 (60.41; 64.91)	61.85 (60.39; 63.35)	0.01 (0.01; 0.01)
54 to 54.9	36.70 (35.58; 37.85)	70.29 (67.79; 72.88)	62.35 (60.87; 63.86)	0.01 (0.01; 0.01)
55 to 55.9	38.86 (37.67; 40.09)	77.41 (74.63; 80.28)	62.97 (61.46; 64.52)	0.01 (0.01; 0.01)
56 to 56.9	40.32 (39.06; 41.62)	83.08 (80.05; 86.23)	63.69 (62.13; 65.28)	0.01 (0.01; 0.01)
57 to 57.9	41.06 (39.72; 42.46)	87.24 (83.90; 90.71)	64.55 (62.93; 66.22)	0.01 (0.01; 0.01)
58 to 58.9	40.94 (39.46; 42.47)	89.33 (85.57; 93.26)	65.60 (63.84; 67.41)	0.01 (0.01; 0.01)
59 to 59.9	39.96 (38.30; 41.69)	89.02 (84.72; 93.53)	66.74 (64.79; 68.76)	0.01 (0.01; 0.01)
60 to 60.9	38.15 (36.26; 40.14)	86.35 (81.39; 91.62)	68.05 (65.80; 70.38)	0.01 (0.01; 0.01)
61 to 61.9	35.37 (33.21; 37.67)	80.97 (75.25; 87.13)	69.65 (66.95; 72.46)	0.01 (0.01; 0.01)
62 to 62.9	32.28 (29.89; 34.87)	74.27 (67.92; 81.22)	71.27 (68.05; 74.65)	0.01 (0.01; 0.01)
63 to 63.9	28.45 (25.87; 31.29)	65.42 (58.59; 73.05)	73.26 (69.31; 77.44)	0.01 (0.01; 0.01)
64 to 64.9	24.52 (21.84; 27.53)	56.00 (48.99; 64.03)	75.42 (70.62; 80.55)	0.01 (0.01; 0.01)
65 to 65.9	20.59 (17.93; 23.66)	46.43 (39.54; 54.52)	77.81 (71.99; 84.09)	0.01 (0.01; 0.02)

AMH: Anti-Mullerian hormone; FSH: follicle-stimulating hormone; LH: luteinizing hormone; SHBG: sex hormone binding globulin

Supplementary Table 7. Comparison between women included and not included in the analyses

	Included in the analyses N=1,608	Excluded from the analyses N=1,885	p-value
Age (years): mean (SD)			
1st clinic	51.0 (4.0)	45.8 (3.2)	<0.001
2nd clinic	53.6 (3.9)	48.1 (3.1)	<0.001
3rd clinic	54.7 (3.8)	49.3 (3.3)	<0.001
4th clinic	56.0 (3.6)	50.3 (3.1)	<0.001
LH (mIU/ml): median (IQR)			
1st clinic	30.7 (12.6; 44.8)	6.6 (4.2; 11.5)	<0.001
2nd clinic	37.3 (28.2; 46.9)	7.6 (4.8; 14.1)	<0.001
3rd clinic	40.1 (31.8; 50.2)	10.8 (5.8; 29.3)	<0.001
4th clinic	37.3 (30.2; 46.2)	16.1 (6.3; 35.7)	<0.001
FSH (mIU/ml): median (IQR)			
1st clinic	53.3 (14.2; 81.0)	6.4 (4.2; 10.8)	<0.001
2nd clinic	69.8 (48.4; 91.2)	7.8 (4.8; 15.9)	<0.001
3rd clinic	83.9 (65.5; 104.1)	12.0 (6.2; 35.6)	<0.001
4th clinic	82.2 (65.4; 101.4)	18.3 (7.5; 57.5)	<0.001
SHBG (nmol/L): median (IQR)			
1st clinic	63.1 (45.4; 86.8)	63.6 (44.4; 87.4)	0.491
2nd clinic	64.5 (47.9; 86.6)	66.2 (48.5; 90.6)	0.011
3rd clinic	71.4 (51.7; 94.4)	76.6 (53.8; 101.3)	<0.001
4th clinic	66.8 (47.4; 89.9)	71.6 (49.4; 94.8)	<0.001
AMH (ng/ml): median (IQR)			
1st clinic	0.01 (0.01; 0.04)	0.40 (0.14; 0.94)	<0.001
2nd clinic	0.01 (0.01; 0.01)	0.16 (0.04; 0.48)	<0.001
3rd clinic	0.01 (0.01; 0.01)	0.09 (0.01; 0.34)	<0.001
4th clinic	0.01 (0.01; 0.01)	0.03 (0.01; 0.19)	<0.001
BMI at first assessment			0.006
Normal *	805 (50.1)	862 (45.8)	
Overweight	536 (33.4)	639 (33.9)	
Obese	266 (16.5)	383 (20.3)	
Smoking status			0.061
Never smoker	803 (54.6)	966 (57.2)	
Former smoker	520 (35.3)	531 (31.5)	
Current smoker	149 (10.1)	191 (11.3)	
Alcohol intake frequency			0.003
Never or less than 4 times a month	449 (40.5)	527 (44.3)	
2 to 3 times a week	367 (33.1)	420 (35.3)	
4 or more times a week	293 (26.4)	243 (20.4)	
Parity			0.369
1	183 (11.4)	207 (11.0)	
2	534 (33.2)	639 (33.9)	
3	396 (24.6)	502 (26.6)	
4+	495 (30.8)	537 (28.5)	
Age at menarche			0.124
Early (≤ 11 years)	240 (16.2)	313 (17.9)	
Average (12-14 years)	1039 (70.0)	1232 (70.4)	
Late (≥ 15 years)	204 (13.8)	204 (11.7)	
Educational achievement			<0.001
CSE / Vocational degree/ O-level	637 (42.3)	994 (56.5)	
A-level	475 (31.6)	475 (27.0)	
University degree	393 (26.1)	291 (16.5)	

A-level: advanced level; BMI: body mass index; CSE: certificate of secondary education; O-level: ordinary level
* 35 women (1.0%) had BMI < 18.5 kg/m^2 (26 with known and 9 with unknown menopause) and were included in the normal BMI category.

Supplementary Table 8. Average predicted means for reproductive hormones across reproductive age, by categories of the lifestyle and reproductive factors.

Hormone	Covariate/ Category	Time since the final menstrual period				
		-5 years	0 years	5 years	10 years	15 years
		Mean (95% CI)	Mean (95% CI)	Mean (95% CI)	Mean (95% CI)	Mean (95% CI)
LH (mIU/ml)	**BMI**					
	Normal	6.38 (5.35; 7.62)	30.21 (27.25; 33.49)	40.61 (36.75; 44.87)	34.21 (30.47; 38.40)	23.19 (19.84; 27.10)
	Overweight	8.61 (7.17; 10.34)	28.18 (25.41; 31.27)	35.45 (32.02; 39.23)	31.27 (27.67; 35.33)	23.38 (19.68; 27.78)
	Obese	8.06 (6.16; 10.54)	23.77 (21.23; 26.60)	30.34 (27.21; 33.84)	28.30 (24.74; 32.38)	22.79 (18.58; 27.95)
	**Smoking**					
	Never smoker	9.03 (7.54; 10.81)	33.68 (29.88; 37.96)	44.05 (39.14; 49.58)	39.00 (34.14; 44.55)	28.78 (24.29; 34.10)
	Former smoker	8.92 (7.25; 10.97)	34.12 (29.88; 38.96)	43.89 (38.55; 49.97)	37.70 (32.68; 43.50)	26.85 (22.35; 32.24)
	Current smoker	6.61 (4.56; 9.58)	31.26 (26.73; 36.55)	43.82 (37.88; 50.68)	38.95 (32.81; 46.24)	27.97 (21.63; 36.17)
	**Alcohol intake**					
	Never or < 4x/month	10.22 (8.34; 12.54)	34.25 (30.75; 38.15)	43.46 (39.17; 48.22)	38.45 (34.02; 43.46)	28.76 (24.33; 34.00)
	2-3x/week	8.21 (6.50; 10.37)	33.33 (29.71; 37.39)	43.82 (39.26; 48.92)	37.91 (33.16; 43.33)	26.98 (22.25; 32.73)
	4+ x/week	7.39 (5.64; 9.68)	33.83 (29.93; 38.25)	46.08 (40.97; 51.82)	39.95 (34.74; 45.94)	28.06 (22.99; 34.23)
	**Parity**					
	1	6.89 (4.98; 9.56)	27.92 (24.44; 31.90)	37.06 (32.69; 42.01)	32.47 (27.92; 37.75)	23.44 (18.76; 29.28)
	2	8.10 (6.65; 9.87)	27.75 (24.96; 30.84)	35.75 (32.25; 39.63)	32.00 (28.15; 36.37)	24.17 (20.03; 29.16)
	3	7.08 (5.73; 8.74)	27.69 (24.86; 30.84)	36.69 (33.08; 40.70)	32.47 (28.58; 36.89)	23.80 (19.73; 28.71)
	4+	6.48 (5.30; 7.93)	27.17 (24.41; 30.23)	34.98 (31.52; 38.81)	29.12 (25.73; 32.97)	19.81 (16.61; 23.63)
	**Age at menarche**					
	Average	7.15 (6.21; 8.24)	28.12 (26.09; 30.30)	36.74 (34.18; 39.49)	31.88 (29.19; 34.83)	22.87 (20.18; 25.91)
	Early	9.52 (7.23; 12.54)	28.58 (25.81; 31.66)	35.69 (32.45; 39.25)	32.16 (28.26; 36.59)	24.89 (20.16; 30.73)
	Late	5.64 (4.25; 7.51)	26.14 (23.51; 29.06)	34.25 (30.91; 37.96)	28.15 (24.11; 32.88)	18.64 (14.26; 24.35)
FSH (mIU/ml)	**BMI**					
	Normal	8.69 (7.23; 10.43)	46.62 (41.44; 52.45)	83.58 (74.52; 93.74)	76.77 (67.30; 87.56)	47.58 (39.73; 56.98)
	Overweight	10.30 (8.51; 12.45)	42.14 (37.43; 47.43)	68.78 (61.20; 77.31)	64.09 (55.78; 73.63)	42.94 (35.18; 52.42)
	Obese	8.39 (6.43; 10.95)	33.32 (29.32; 37.88)	56.16 (49.49; 63.73)	55.72 (47.84; 64.90)	40.43 (31.96; 51.14)

	**Smoking**					
	Never smoker	12.09 (9.98; 14.65)	55.74 (48.61; 63.91)	96.57 (84.35; 110.58)	91.86 (78.95; 106.88)	61.37 (50.44; 74.66)
	Former smoker	12.54 (10.09; 15.60)	57.32 (49.21; 66.77)	96.91 (83.40; 112.6)	89.39 (75.93; 105.23)	57.73 (46.77; 71.26)
	Current smoker	9.07 (6.27; 13.12)	48.19 (40.40; 57.50)	88.72 (74.83; 105.19)	85.12 (69.97; 103.55)	55.62 (41.33; 74.86)
	**Alcohol intake**					
	Never or < 4x/month	13.89 (11.34; 17.00)	57.41 (50.75; 64.93)	94.65 (84.04; 106.61)	89.00 (77.51; 102.20)	60.13 (49.68; 72.79)
	2-3x/week	11.55 (9.17; 14.55)	56.57 (49.72; 64.38)	98.07 (86.47; 111.23)	90.27 (77.52; 105.11)	57.25 (45.66; 71.80)
	4+ x/week	10.55 (8.19; 13.60)	56.10 (48.81; 64.47)	102.62 (89.68; 117.43)	97.56 (83.25; 114.33)	63.07 (50.15; 79.33)
	**Parity**					
	1	9.20 (6.67; 12.69)	40.95 (35.15; 47.71)	69.75 (60.31; 80.67)	65.98 (55.52; 78.40)	44.14 (34.21; 56.96)
	2	9.08 (7.41; 11.12)	40.96 (36.28; 46.26)	71.07 (63.07; 80.08)	68.53 (59.21; 79.31)	46.74 (37.50; 58.26)
	3	9.67 (7.78; 12.02)	42.33 (37.41; 47.90)	71.07 (63.04; 80.13)	66.47 (57.48; 76.86)	44.05 (35.39; 54.85)
	4+	7.52 (6.12; 9.25)	39.00 (34.50; 44.09)	67.66 (59.99; 76.33)	60.41 (52.43; 69.61)	36.52 (29.73; 44.85)
	**Age at menarche**					
	Average	9.29 (8.02; 10.76)	42.17 (38.70; 45.95)	71.28 (65.58; 77.47)	65.96 (59.64; 72.96)	42.84 (37.07; 49.50)
	Early	9.76 (7.42; 12.85)	40.53 (36.01; 45.62)	68.14 (61.05; 76.06)	65.74 (56.78; 76.12)	45.72 (35.78; 58.43)
	Late	5.94 (4.47; 7.90)	38.68 (34.28; 43.66)	72.14 (64.01; 81.29)	63.03 (52.75; 75.31)	35.28 (25.48; 48.86)
SHBG (nmol /ml)	**BMI**					
	Normal	81.98 (73.65; 91.26)	75.97 (69.99; 82.47)	75.85 (70.07; 82.12)	78.93 (72.22; 86.25)	84.08 (74.98; 94.28)
	Overweight	59.38 (53.23; 66.25)	55.98 (51.57; 60.77)	56.74 (52.39; 61.45)	59.77 (54.52; 65.52)	64.33 (56.84; 72.82)
	Obese	53.31 (46.35; 61.32)	45.08 (41.35; 49.15)	44.48 (40.93; 48.34)	47.86 (43.36; 52.82)	54.11 (46.84; 62.51)
	**Smoking**					
	Never smoker	112.73 (100.34; 126.66)	98.04 (89.11; 107.86)	97.6 (88.88; 107.17)	104.73 (94.55; 116.00)	117.30 (103.47; 132.99)
	Former smoker	95.48 (83.89; 108.67)	95.45 (85.88; 106.09)	97.31 (87.76; 107.91)	100.07 (89.55; 111.83)	103.38 (90.28; 118.39)
	Current smoker	103.76 (85.89; 125.37)	96.04 (85.29; 108.14)	95.61 (85.37; 107.07)	99.13 (87.18; 112.73)	105.21 (87.75; 126.15)
	**Alcohol intake**					
	Never or < 4x/month	109.29 (97.84; 122.09)	96.30 (88.67; 104.58)	95.68 (88.24; 103.74)	101.64 (92.67; 111.48)	112.19 (99.30; 126.76)
	2-3x/week	96.88 (85.84; 109.34)	92.61 (84.89; 101.04)	93.90 (86.08; 102.43)	98.20 (88.59; 108.85)	104.51 (90.69; 120.43)
	4+ x/week	96.98 (84.91; 110.77)	92.04 (83.77; 101.11)	92.66 (84.51; 101.60)	96.32 (86.60; 107.12)	101.94 (88.23; 117.77)
	**Parity**					

	1	56.25 (47.32; 66.89)	54.82 (49.08; 61.24)	58.03 (52.27; 64.42)	63.90 (56.64; 72.08)	71.89 (60.72; 85.10)
	2	66.83 (59.19; 75.46)	59.40 (54.24; 65.05)	58.45 (53.53; 63.83)	60.95 (55.02; 67.52)	65.70 (57.03; 75.70)
	3	60.87 (53.68; 69.01)	57.79 (52.72; 63.35)	59.90 (54.85; 65.42)	64.92 (58.62; 71.90)	72.12 (62.63; 83.05)
	4+	61.94 (54.83; 69.98)	56.35 (51.45; 61.71)	54.84 (50.21; 59.89)	55.55 (50.23; 61.44)	57.61 (50.33; 65.95)
	**Age at menarche**					
	Normal	60.17 (55.08; 65.74)	56.53 (53.08; 60.21)	57.00 (53.67; 60.52)	59.69 (55.57; 64.12)	63.87 (57.92; 70.43)
	Early	63.40 (55.17; 72.85)	55.64 (51.21; 60.46)	54.70 (50.61; 59.12)	57.35 (51.93; 63.34)	62.40 (53.47; 72.82)
	Late	68.81 (59.54; 79.52)	58.64 (53.78; 63.94)	58.09 (53.44; 63.14)	62.58 (55.82; 70.16)	70.75 (58.59; 85.43)
AMH (ng/ml)	**BMI**					
	Normal	0.19 (0.15; 0.25)	0.02 (0.01; 0.02)	0.01 (0.01; 0.01)	0.01 (0.01; 0.02)	0.02 (0.02; 0.03)
	Overweight	0.10 (0.08; 0.13)	0.02 (0.01; 0.02)	0.01 (0.01; 0.01)	0.01 (0.01; 0.02)	0.02 (0.02; 0.02)
	Obese	0.10 (0.07; 0.14)	0.02 (0.02; 0.02)	0.01 (0.01; 0.02)	0.01 (0.01; 0.02)	0.02 (0.01; 0.02)
	**Smoking**					
	Never smoker	0.11 (0.09; 0.14)	0.02 (0.01; 0.02)	0.01 (0.01; 0.01)	0.01 (0.01; 0.02)	0.02 (0.02; 0.02)
	Former smoker	0.15 (0.11; 0.19)	0.02 (0.01; 0.02)	0.01 (0.01; 0.01)	0.01 (0.01; 0.02)	0.02 (0.02; 0.03)
	Current smoker	0.21 (0.12; 0.37)	0.02 (0.01; 0.02)	0.01 (0.01; 0.01)	0.01 (0.01; 0.02)	0.02 (0.01; 0.03)
	**Alcohol intake**					
	Never or < 4x/month	0.11 (0.08; 0.14)	0.02 (0.01; 0.02)	0.01 (0.01; 0.01)	0.01 (0.01; 0.02)	0.02 (0.02; 0.02)
	2-3x/week	0.13 (0.09; 0.18)	0.02 (0.01; 0.02)	0.01 (0.01; 0.01)	0.01 (0.01; 0.02)	0.02 (0.02; 0.03)
	4+ x/week					
	**Parity**	0.19 (0.13; 0.28)	0.02 (0.01; 0.02)	0.01 (0.01; 0.01)	0.01 (0.01; 0.02)	0.02 (0.02; 0.03)
	1	0.21 (0.14; 0.34)	0.02 (0.01; 0.02)	0.01 (0.01; 0.01)	0.01 (0.01; 0.02)	0.02 (0.02; 0.03)
	2	0.11 (0.09; 0.14)	0.02 (0.01; 0.02)	0.01 (0.01; 0.01)	0.01 (0.01; 0.02)	0.02 (0.02; 0.02)
	3	0.11 (0.08; 0.14)	0.02 (0.01; 0.02)	0.01 (0.01; 0.01)	0.01 (0.01; 0.02)	0.02 (0.02; 0.02)
	4+	0.17 (0.13; 0.23)	0.02 (0.01; 0.02)	0.01 (0.01; 0.01)	0.02 (0.01; 0.02)	0.02 (0.02; 0.03)
	**Age at menarche**					
	Average	0.14 (0.12; 0.17)	0.02 (0.01; 0.02)	0.01 (0.01; 0.01)	0.01 (0.01; 0.02)	0.02 (0.02; 0.02)
	Early	0.11 (0.08; 0.17)	0.02 (0.01; 0.02)	0.01 (0.01; 0.01)	0.02 (0.01; 0.02)	0.02 (0.02; 0.03)
	Late	0.12 (0.08; 0.17)	0.02 (0.01; 0.02)	0.01 (0.01; 0.01)	0.01 (0.01; 0.02)	0.02 (0.01; 0.03)

Predictions are made for a woman aged 51 years. AMH: Anti-Mullerian hormone; FSH: follicle-stimulating hormone; LH: luteinizing hormone; SHBG: sex hormone binding globulin

Supplementary Table 9. P-values for effect modification in the hormone trajectories across reproductive age.

	BMI	Smoking	Alcohol intake	Parity	Age at menarche
Models with imputed data (n=1,608)					
LH	<0.001	0.503	0.206	0.545	0.177
FSH	0.011	0.725	0.202	0.668	0.066
SHBG	0.378	0.019	0.507	0.023	0.437
AMH	<0.001	0.040	0.172	0.019	0.451
Models with complete data on risk factors and confounders					
	n = 1,042	n = 1,097	n = 1,097	n = 1,042	n = 1,410
LH	<0.001	0.678	0.093	0.724	0.118
FSH	0.004	0.695	0.125	0.415	0.010
SHBG	0.893	0.002	0.210	0.292	0.478
AMH	<0.001	0.358	0.262	0.001	0.165

AMH: Anti-Mullerian hormone; BMI: body mass index; FSH: follicle-stimulating hormone; LH: luteinizing hormone; SHBG: sex hormone binding globulin

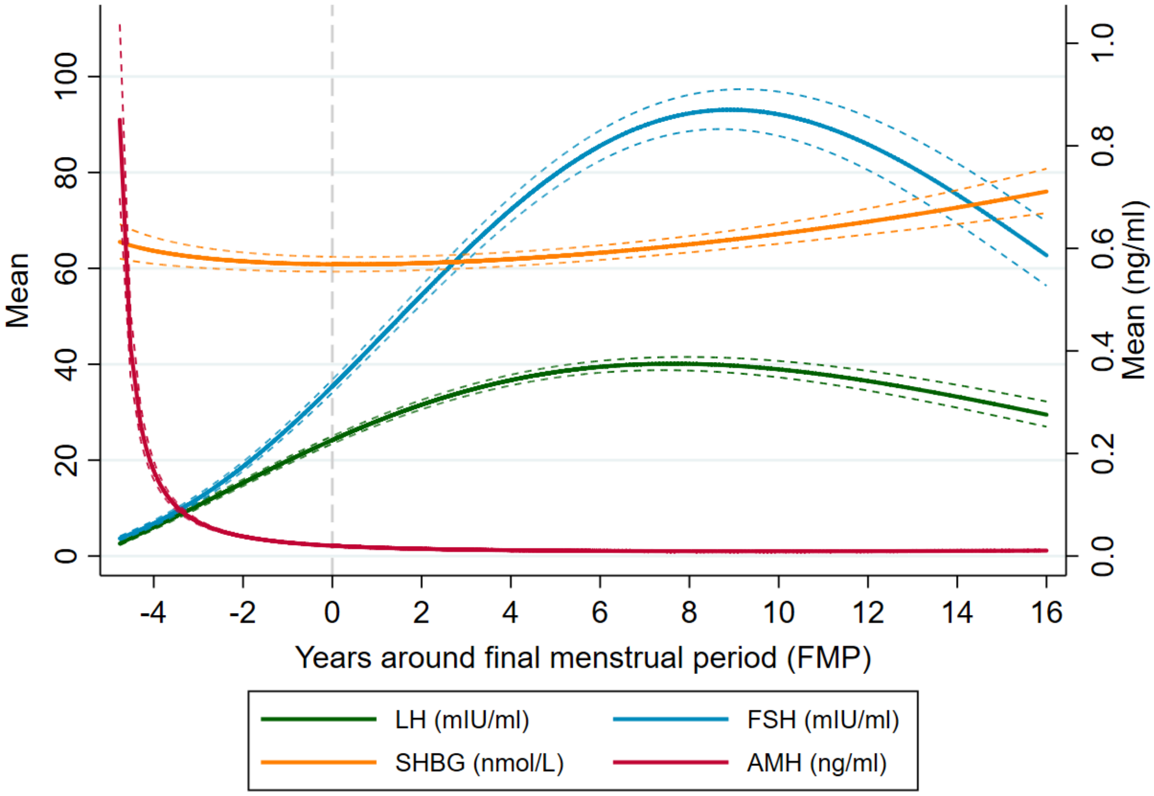

Supplementary Figure 1. Unadjusted average predicted population means (95% CI) for reproductive hormones across reproductive age (n=1,608).

Dashed vertical line corresponds to menopause.

A)

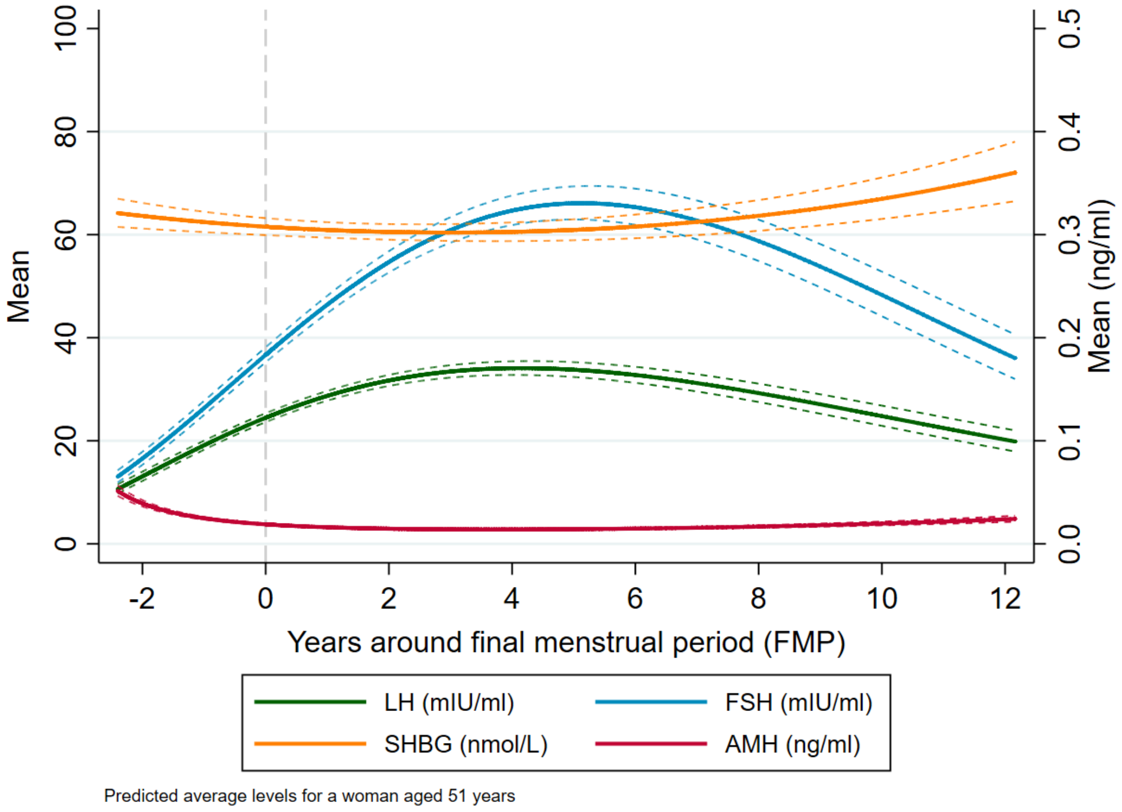

B)

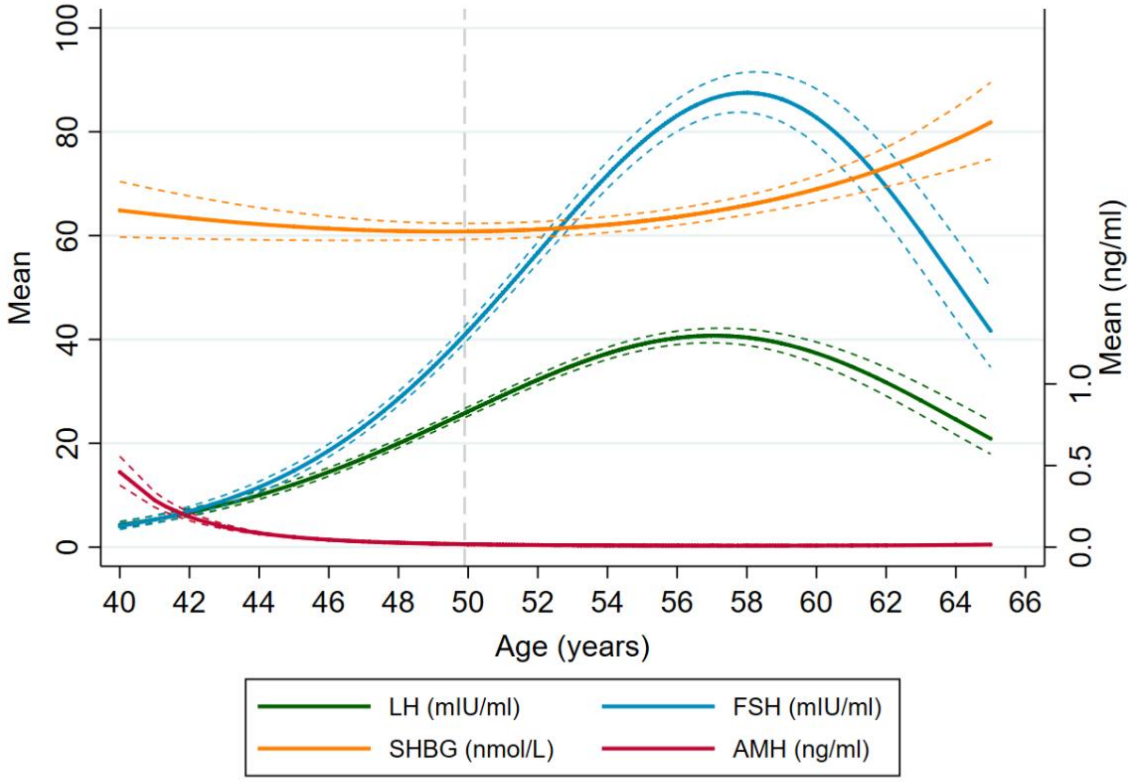

Supplementary Figure 2. Average predicted population means (95% CI) for reproductive hormones across reproductive age (A) and chronological age (B), restricted at the 5th and 95th centiles of age at final menstrual period (n=1,552).

Dashed vertical line corresponds to menopause (A) or average age of menopause in the study (B).

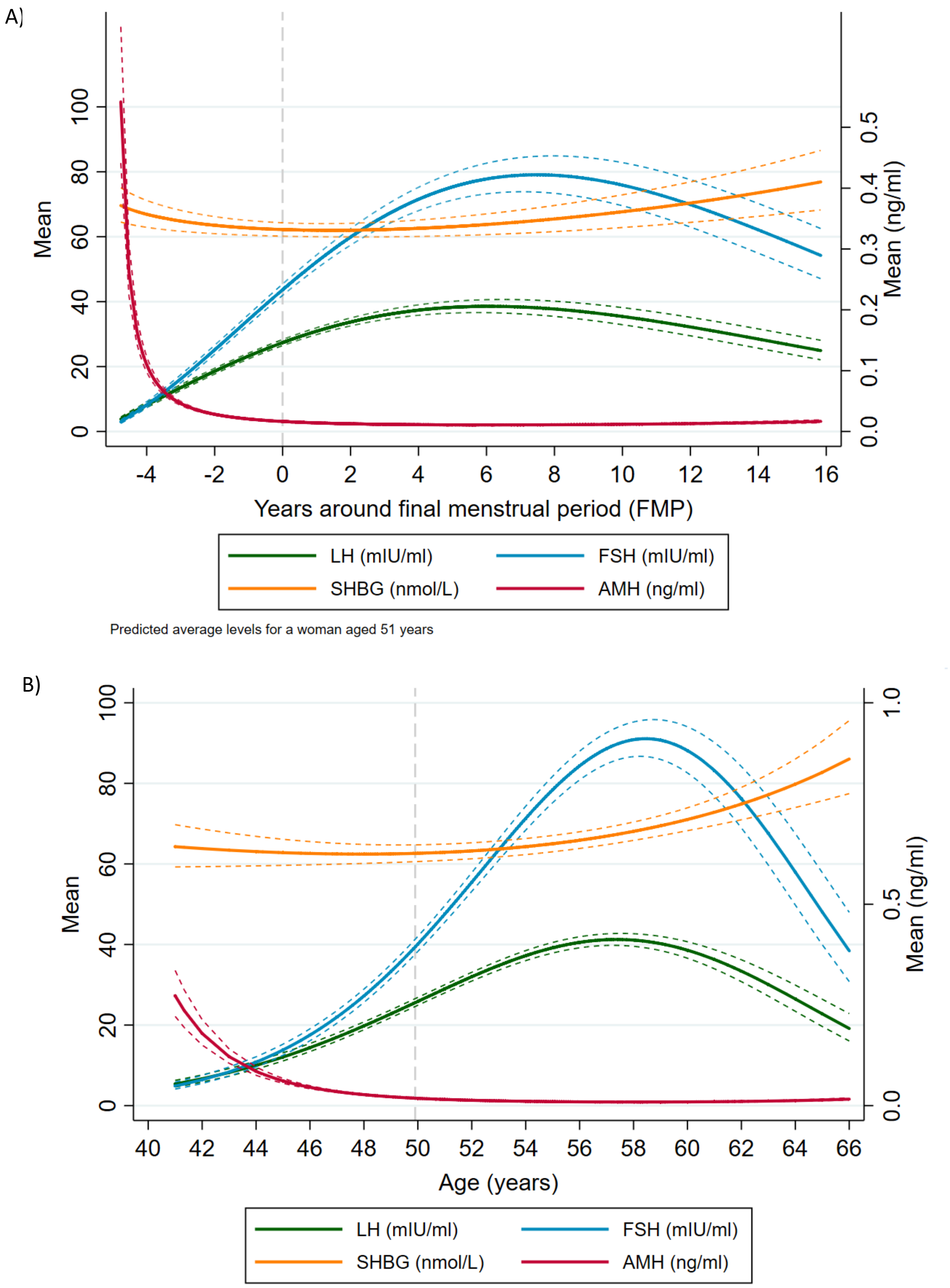

Supplementary Figure 3. Average predicted population means (95% CI) for reproductive hormones across reproductive age (A) and chronological age (B) in women with 3 or 4 repeat measures (n=818).

Dashed vertical line corresponds to menopause (A) or average age of menopause in the study (B).

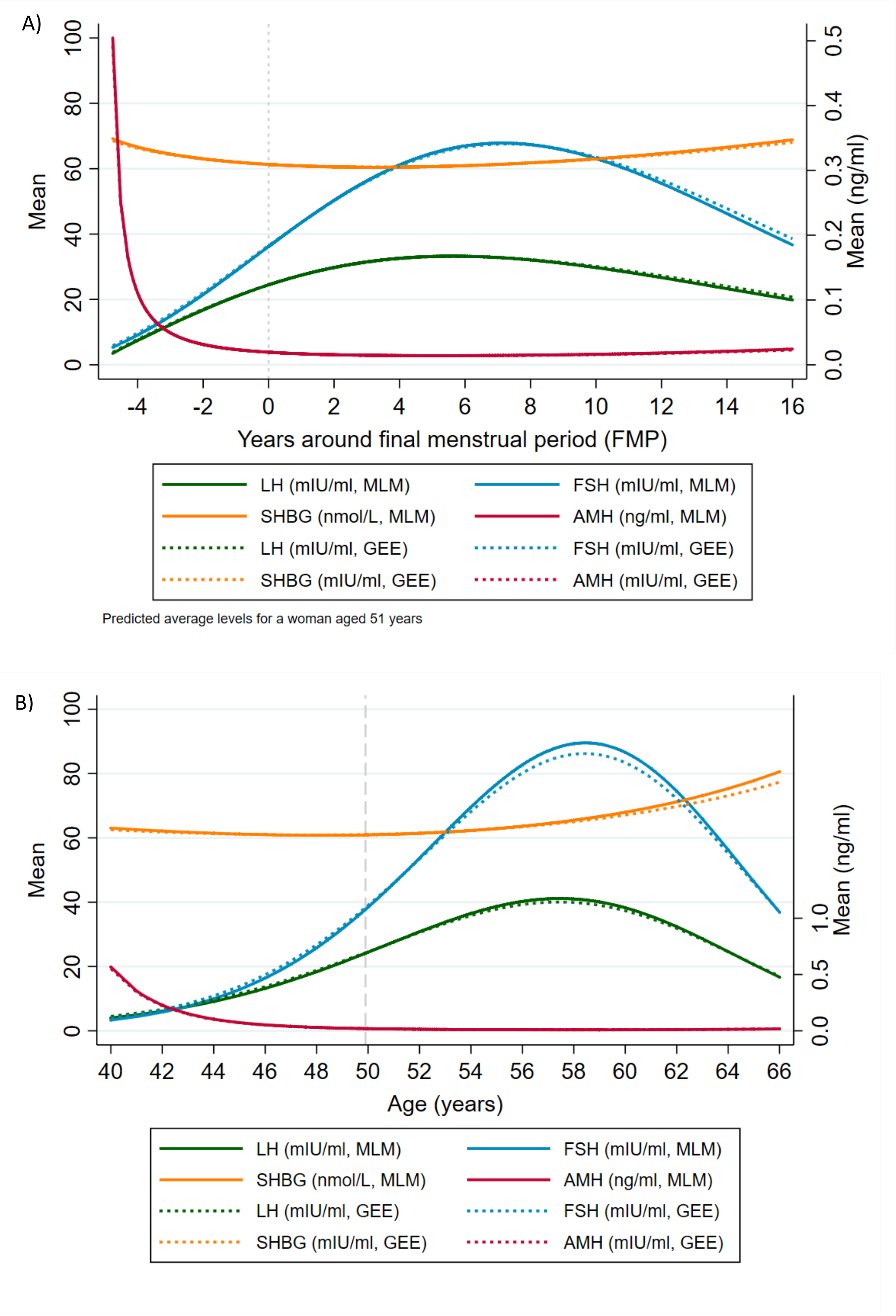

Supplementary Figure 4. Average predicted population means for reproductive hormones across reproductive age (A) and chronological age (B) estimated using multilevel models (MLM) and generalised estimating equations (GEE) (n=1,608).

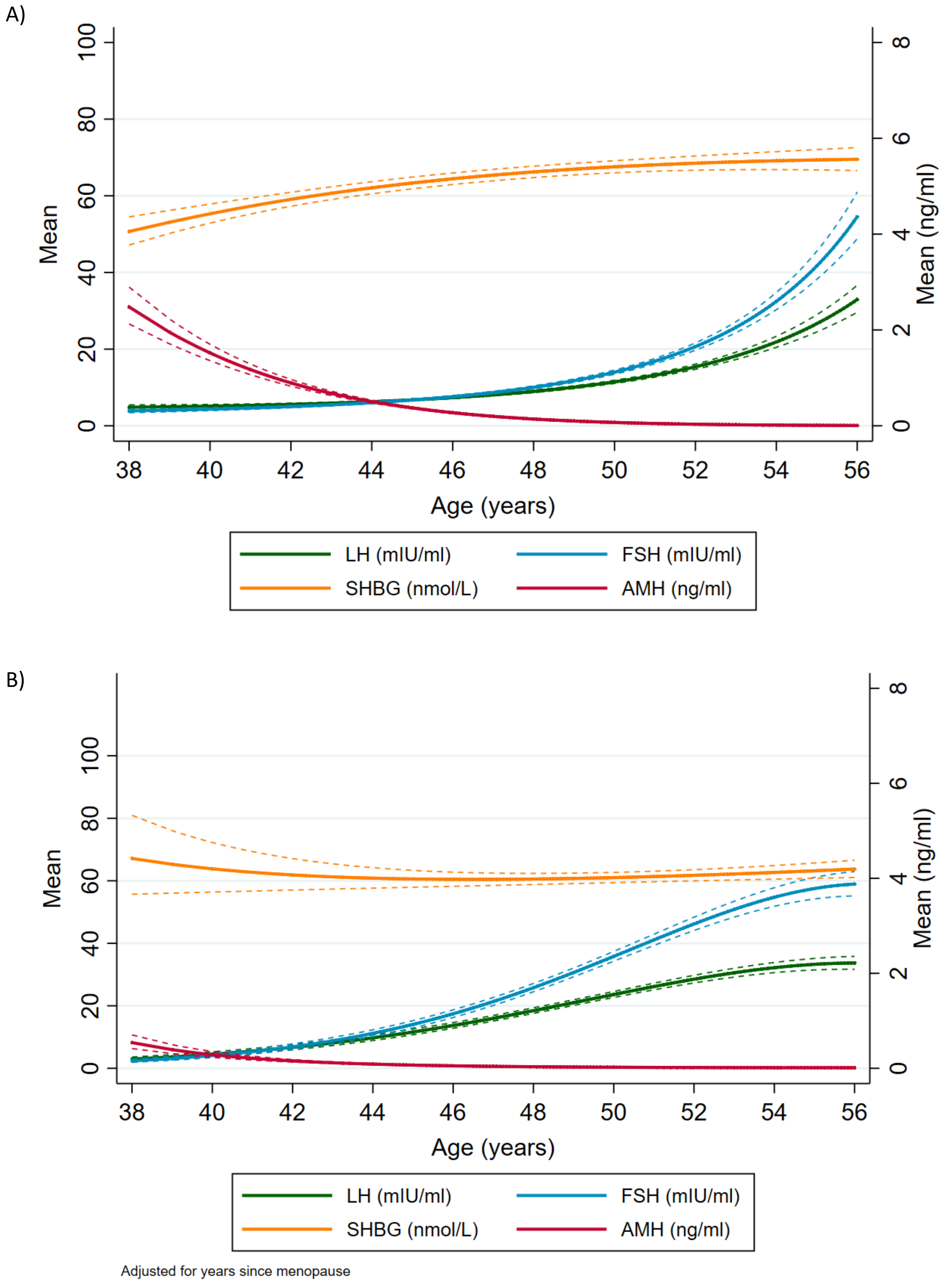

Supplementary Figure 5. Average predicted population means (95% CI) for reproductive hormones across chronological age in women excluded from the main analyses due to not reaching menopause or to insufficient data (A) and in women included in the main analyses, i.e., who reached menopause (B) (n= 1,885 and n= 1,608, respectively).

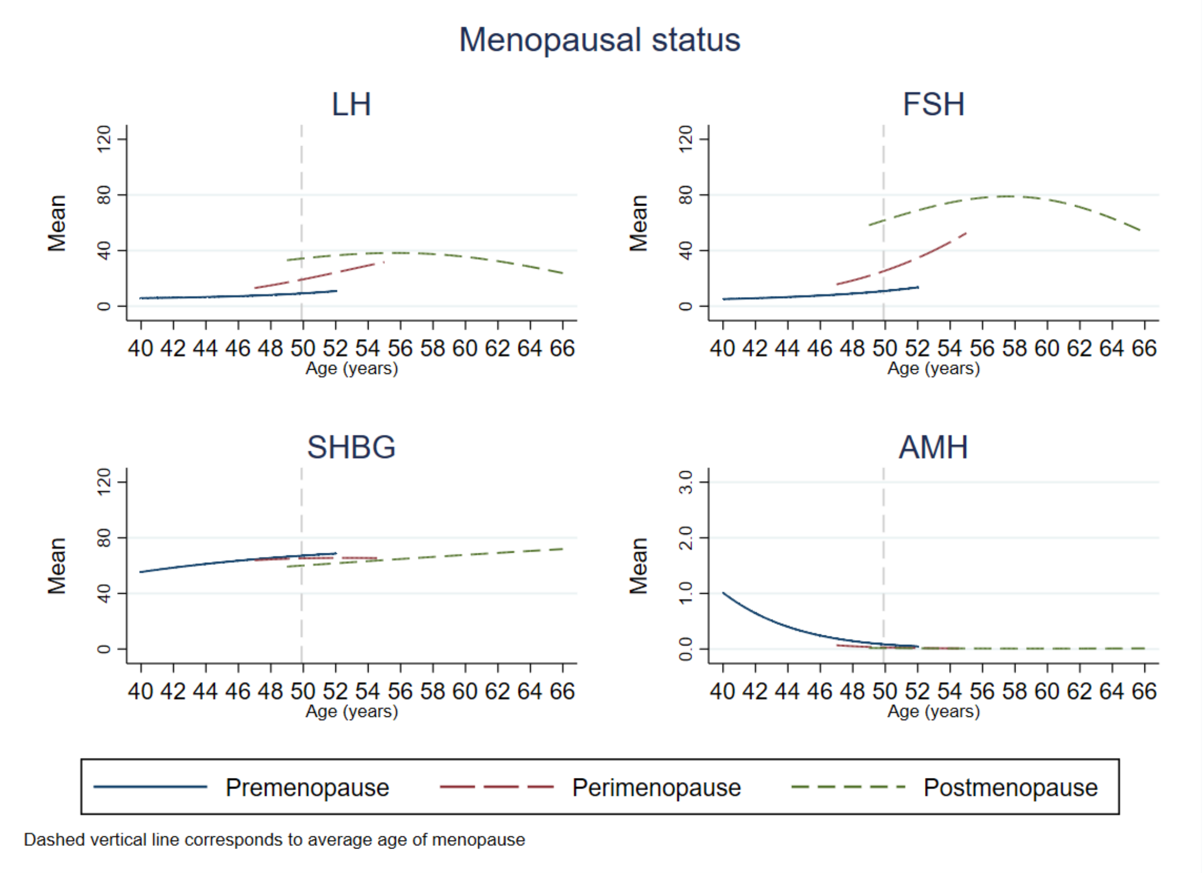

Supplementary Figure 6. Generalized estimating equations (GEE) average predicted population means for reproductive hormones across chronological age by menopausal stages (n= 3,460).

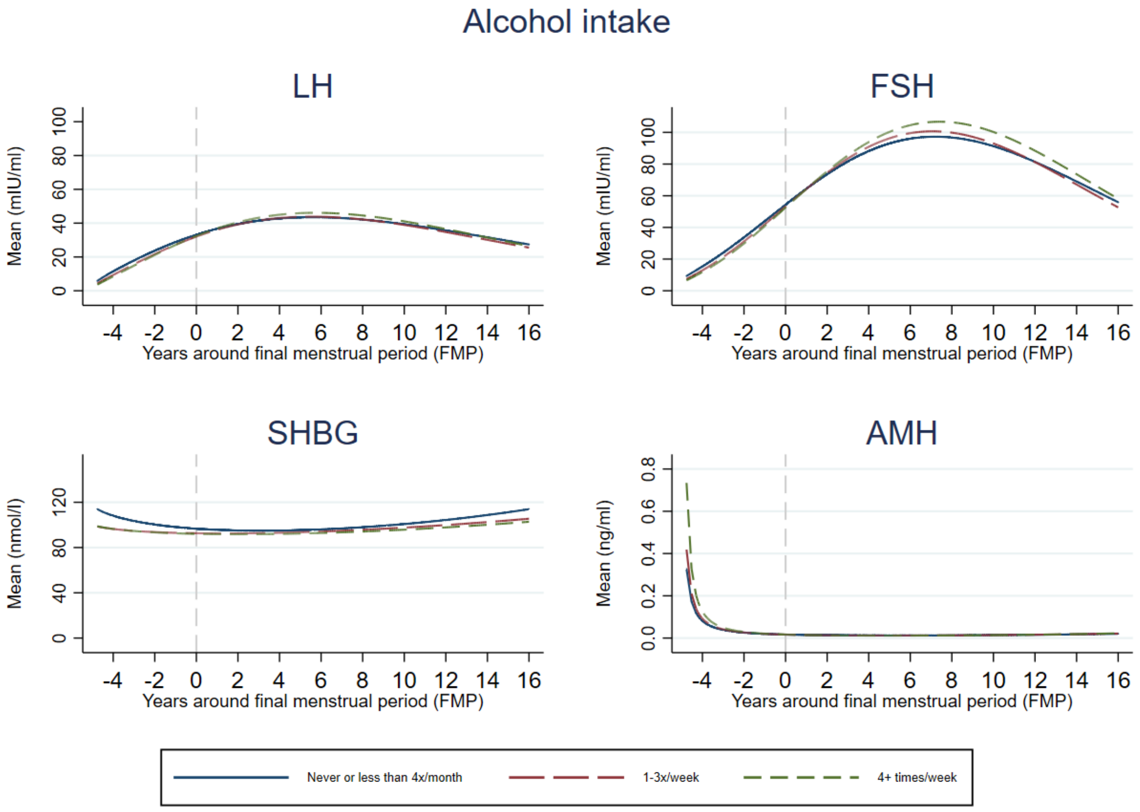

Adjusted for age, educational achievement, BMI and smoking status

Supplementary Figure 7. Association of alcohol intake with mean hormone levels for females at different reproductive age (n= 1,608).

In these analyses, all covariates used in the adjustment were set to the mean value or the reference category: age (51 years), education (CSE/ Vocational degree/ O-level), body mass index (<25 kg/m^2), smoking status (never smoker), alcohol intake (never or less than 4 times a month), age at menarche (13 years).

A)

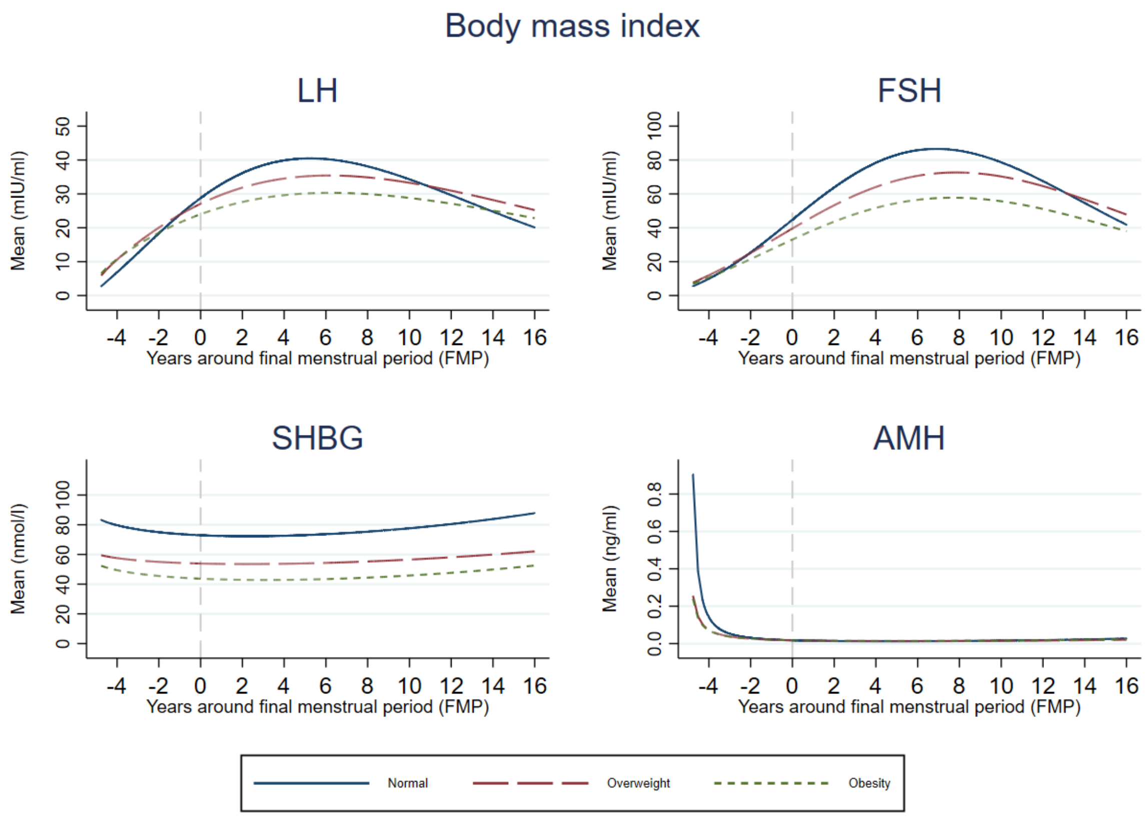

Adjusted for age, educational achievement, age at menarche, smoking status and alcohol intake

B)

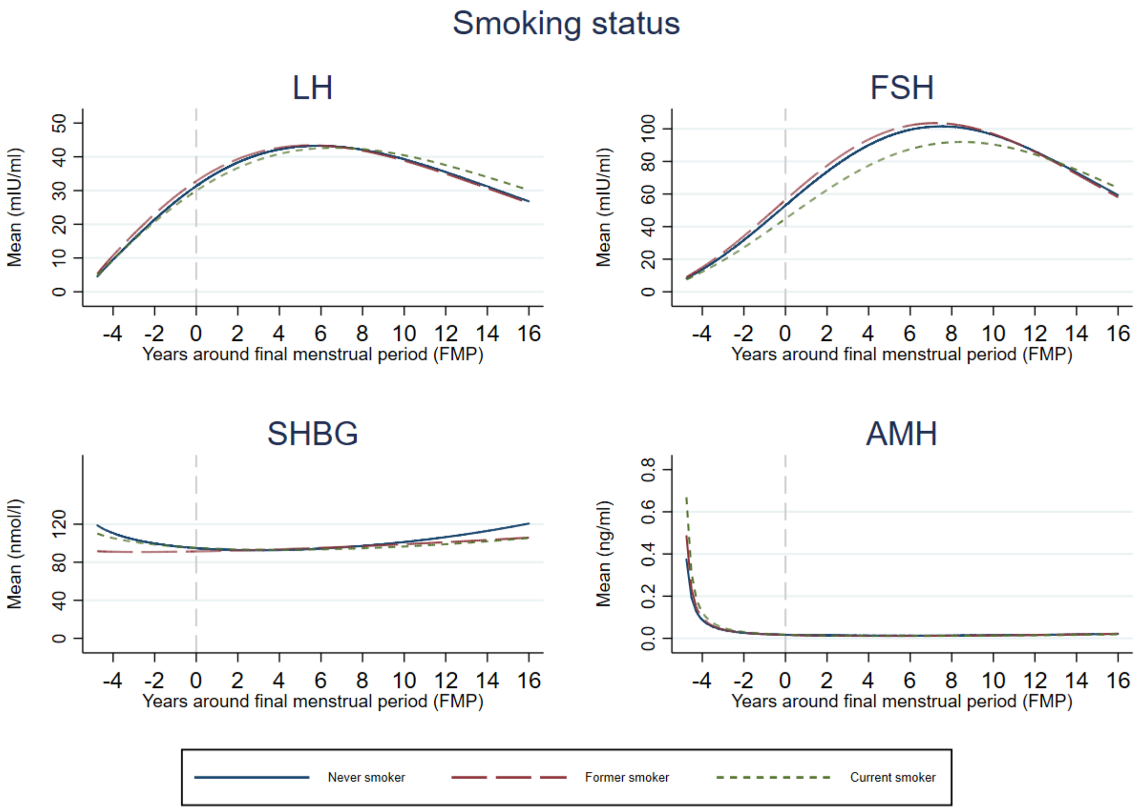

Adjusted for age, educational achievement, BMI and alcohol intake

C) Alcohol intake

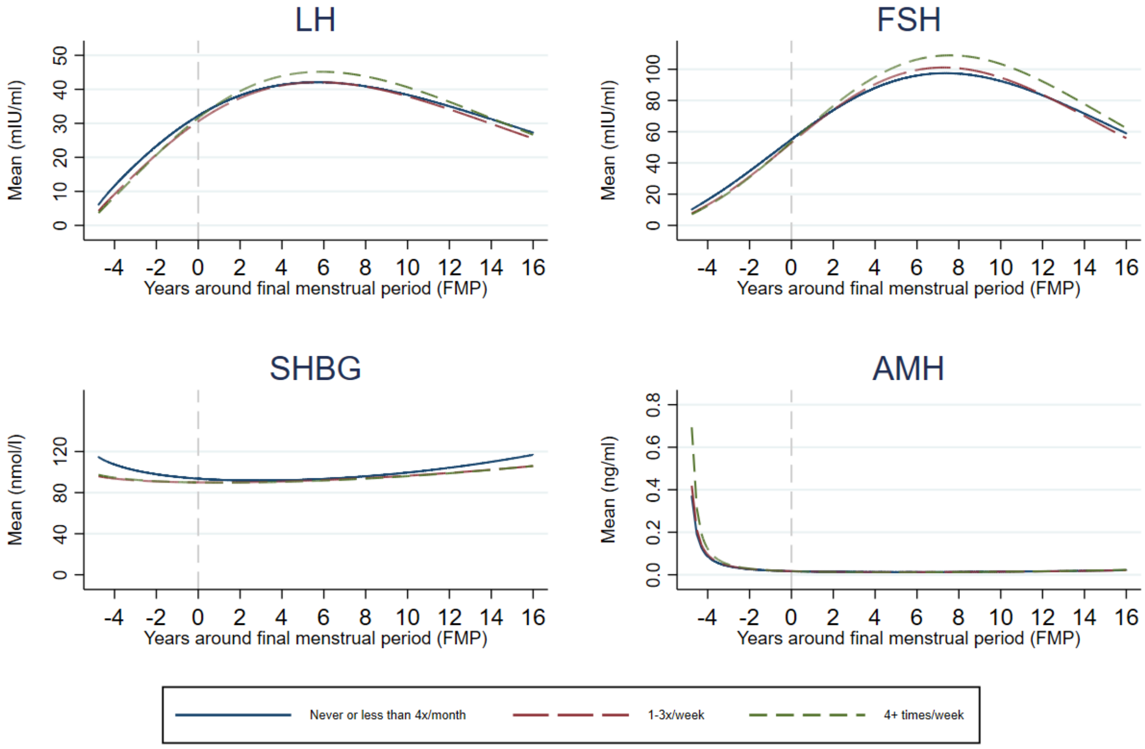

Adjusted for age, educational achievement, BMI and smoking status

D) Parity

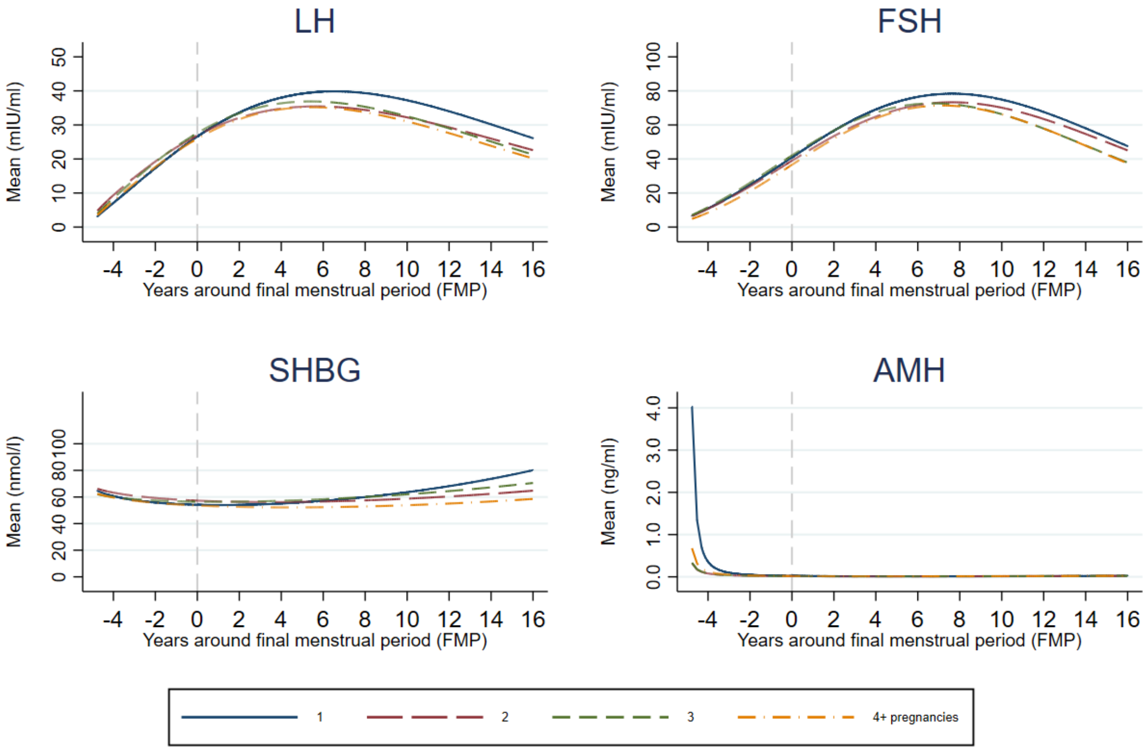

Adjusted for age, educational achievement, age at menarche, smoking status and alcohol intake

E)

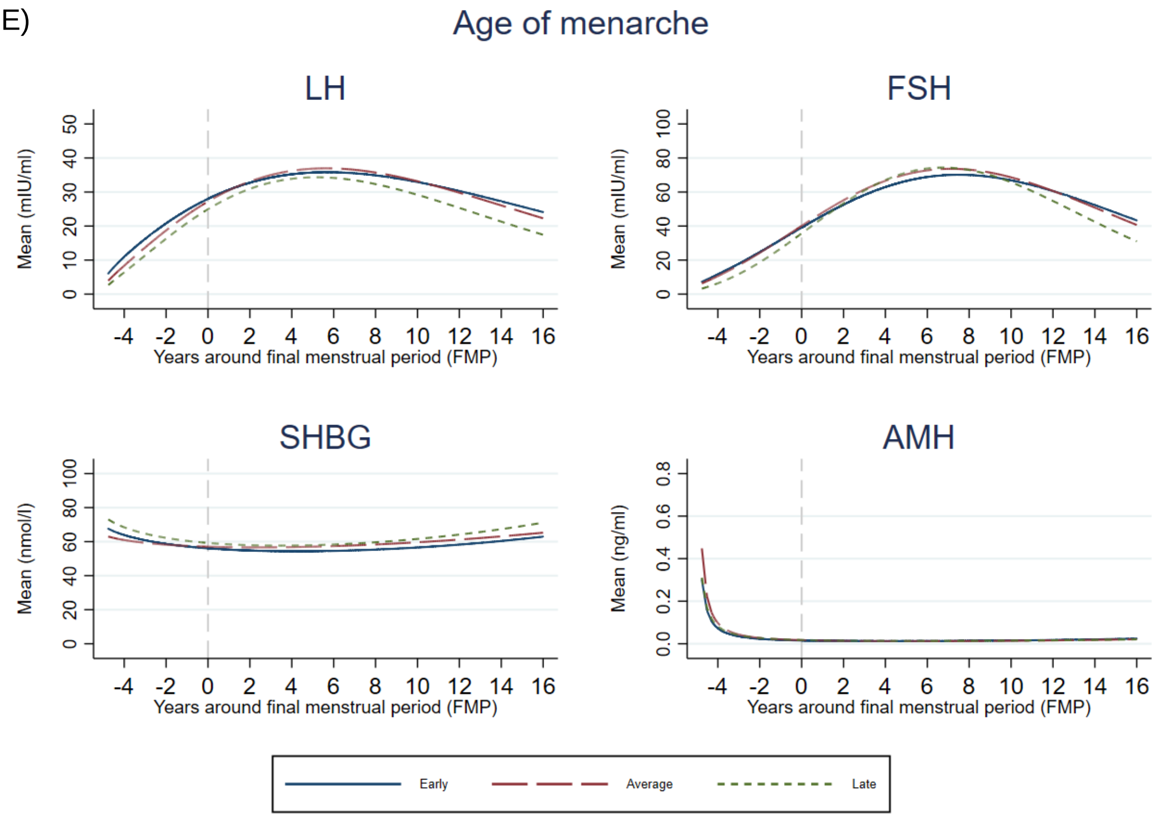

Adjusted for age and educational achievement

Supplementary Figure 8. Association of lifestyle and reproductive factors with mean hormone levels for females at different reproductive age in women with complete data in all covariates (n = 1,042 – 1,410).

In these analyses, all covariates used in the adjustment were set to the mean value or the reference category: age (51 years), education (CSE/ Vocational degree/ O-level), body mass index (<25 kg/m^2), smoking status (never smoker), alcohol intake (never or less than 4 times a month), age at menarche (13 years).

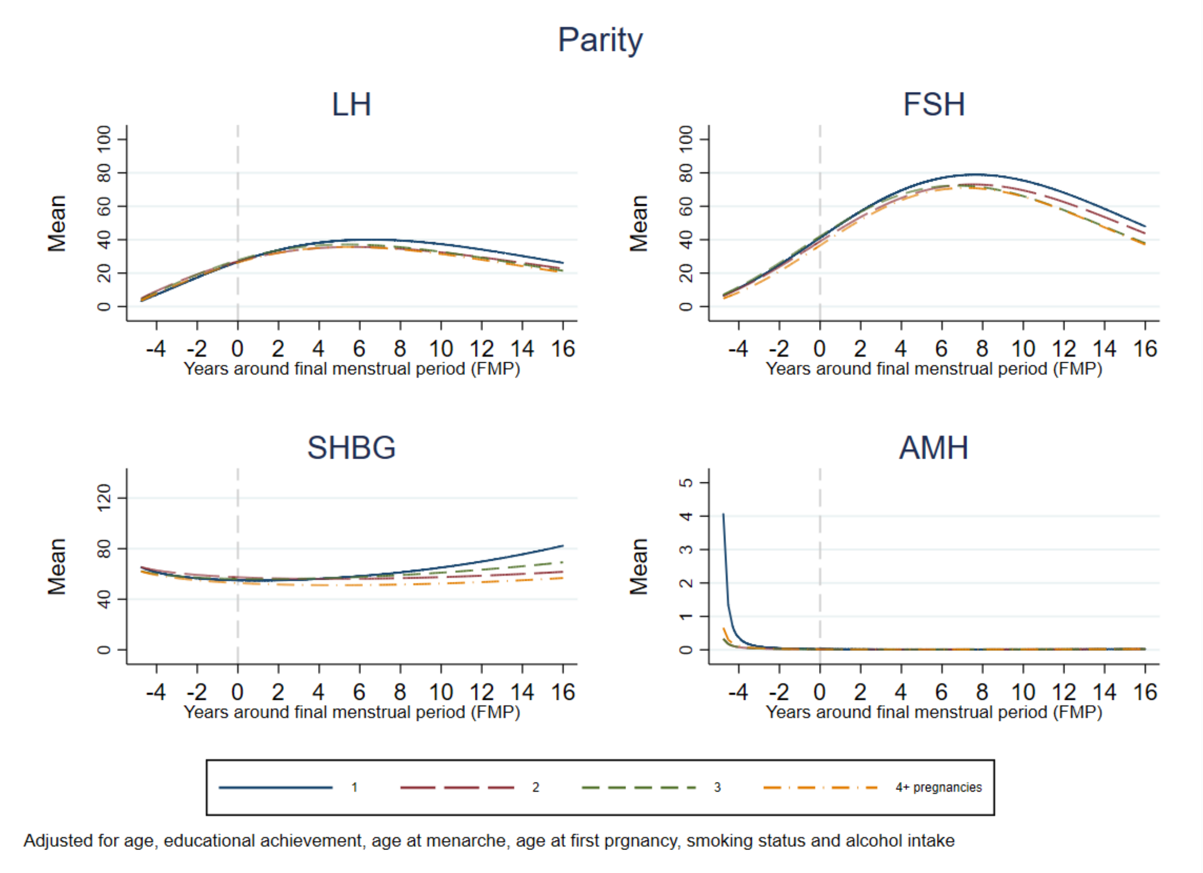

Supplementary Figure 9. Association of parity with mean hormone levels for females at different reproductive age in women with complete data in all covariates (n = 1,037).

In these analyses, all covariates used in the adjustment were set to the mean value or the reference category: age (51 years), education (CSE/ Vocational degree/ O-level), age at menarche (13 years), age at first pregnancy (28.2 years), smoking status (never smoker), alcohol intake (never or less than 4 times a month).
